# Elucidating the microbiome of the sustainable peat replacers composts and nature management residues

**DOI:** 10.3389/fmicb.2022.983855

**Published:** 2022-09-26

**Authors:** Steffi Pot, Caroline De Tender, Sarah Ommeslag, Ilse Delcour, Johan Ceusters, Bart Vandecasteele, Jane Debode, Karen Vancampenhout

**Affiliations:** ^1^Division Forest, Nature and Landscape, Department of Earth and Environmental Sciences, KU Leuven, Geel, Belgium; ^2^Plant Sciences Unit, Flanders Research Institute for Agriculture, Fisheries and Food (ILVO), Merelbeke, Belgium; ^3^Department of Plant Biotechnology and Bioinformatics, Ghent University, Zwijnaarde, Belgium; ^4^PCS Ornamental Plant Research, Destelbergen, Belgium; ^5^Division of Crop Biotechnics, Department of Biosystems, Research Group for Sustainable Crop Production & Protection, KU Leuven, Geel, Belgium; ^6^Centre for Environmental Sciences, Environmental Biology, UHasselt, Diepenbeek, Belgium

**Keywords:** microbiology, composts, Biolog EcoPlates, PLFA analysis, sustainable horticultural substrates, nature management residues, metabarcoding

## Abstract

Sustainable peat alternatives, such as composts and management residues, are considered to have beneficial microbiological characteristics compared to peat-based substrates. Studies comparing microbiological characteristics of these three types of biomass are, however, lacking. This study examined if and how microbiological characteristics of subtypes of composts and management residues differ from peat-based substrates, and how feedstock and (bio)chemical characteristics drive these characteristics. In addition, microbiome characteristics were evaluated that may contribute to plant growth and health. These characteristics include: genera associated with known beneficial or harmful microorganisms, microbial diversity, functional diversity/activity, microbial biomass, fungal to bacterial ratio and inoculation efficiency with the biocontrol fungus *Trichoderma harzianum*. Bacterial and fungal communities were studied using 16S rRNA and ITS2 gene metabarcoding, community-level physiological profiling (Biolog EcoPlates) and PLFA analysis. Inoculation with *T. harzianum* was assessed using qPCR. Samples of feedstock-based subtypes of composts and peat-based substrates showed similar microbial community compositions, while subtypes based on management residues were more variable in their microbial community composition. For management residues, a classification based on pH and hemicellulose content may be relevant for bacterial and fungal communities, respectively. Green composts, vegetable, fruit and garden composts and woody composts show the most potential to enhance plant growth or to suppress pathogens for non-acidophilic plants, while grass clippings, chopped heath and woody fractions of compost show the most potential for blends for calcifuge plants. Fungal biomass was a suitable predictor for inoculation efficiency of composts and management residues.

## Introduction

In horticulture, peat is a major constituent of diverse substrates. Its low pH, low bulk density, optimal EC, high porosity, high water holding capacity and homogeneity make peat an ideal substrate for growing many ornamental plants (Schmilewski, 2008; Michel, 2010). However, environmental concerns regarding peat extraction and utilization are rapidly growing. Peatlands are valuable habitats for protected animal and plant species, are important carbon sinks, and provide environmental services, such as regulation of local water quality and flood protection (Alexander et al., 2008). Moreover, draining of peatlands and extraction of peat accelerates peat decomposition to such an extent that peatlands become a major source of greenhouse gasses (Bonn et al., 2016).

Hence, there is an urgent need to find sustainable alternatives for peat in horticulture. A promising avenue in the search for more sustainable peat alternatives may be the use of residual biomass, such as composts and nature management residues. Studies have shown that composts can have physicochemical and (bio)chemical properties that make them suitable peat alternatives for multiple types of plants (Hernandez-Apaolaza et al., 2005; Bustamante et al., 2008; Herrera et al., 2008; Vandecasteele et al., 2021). Management residues, such as sods and chopped biomass from heathland management efforts, can replace 40% of peat in growing media for calcifuge ornamental plants without loss of plant quality (Miserez et al., 2019a).

Apart from supporting plant growth, horticultural substrates also provide a habitat for microorganisms. The interaction between plants and their rhizosphere microbiome can be beneficial and even critical to plant health, growth and productivity (Chaparro et al., 2012; Quiza et al., 2015). Rhizosphere microorganisms can improve nutrient availability, reduce biotic and abiotic stress, and increase plant defenses (Figueiredo et al., 2011). Microbial communities in the rhizosphere can contribute to the reduction of biotic stress and the suppression of plant pathogens by several types of interaction between microorganisms and pathogens, including competition for nutrients and ecological niches, antibiosis, predation, parasitism, and the activation of disease resistance in plants (Ntougias et al., 2008). Various rhizosphere microorganisms are known for their beneficial effects on plant growth and health, including nitrogen-fixing bacteria, mycorrhizal fungi, plant growth promoting rhizobacteria (PGPR) and fungi (PGPF), and biocontrol agents (Berendsen et al., 2012). Beneficial microorganisms present in horticultural substrates may thus contribute positively to the rhizosphere microbiome and enhance plant growth and resistance to plant pathogens. Additionally, substrates with higher general microbial biomass or diversity may be less susceptible for colonization by other organisms due to stronger competition for nutrients and niches, and may therefore be more suppressive to pathogens (Chaparro et al., 2012; Bongiorno et al., 2019). Studies have also shown a positive effect of microbial biomass and diversity on plant growth and productivity (Wagg et al., 2011; Weidner et al., 2015; Shen et al., 2016; Kolton et al., 2017). Higher metabolic activity and functional diversity can be associated with disease suppression and plant growth promotion (Brussaard et al., 2007; Mendes et al., 2011; Alam et al., 2014; Kolton et al., 2017; Neher et al., 2022). However, horticultural substrates may also harbor potential plant or human pathogens, which poses a risk for plant and human health, but also for the environment (Cartwright, 1995; Waller et al., 2008; Al-Sadi et al., 2011, 2016).

Despite their importance in terms of plant growth and health, the microbiological characteristics of peat alternatives have not received much attention in scientific literature. The current understanding of the microbial communities in peat-based substrates and peat alternatives, such as composts and management residues, is still limited. It is assumed that peat does not provide a suitable food base for microorganisms to grow as it has a high amount of strongly polymerized organic matter, and therefore a low energy reserve (Hoitink and Boehm, 1999). Hence, peat is often considered as an ineffective medium to harbor (beneficial) microorganisms and to support sustained biological control (Hoitink and Boehm, 1999; Krause et al., 2001), yet data to support such assumption are few. Peat alternatives are assumed to be more suitable media for (beneficial) microorganisms because of the higher amount of available energy reserves. Composts and management residues have been shown to have a higher microbial biomass than peat (Vandecasteele et al., 2021), and are expected to have a higher diversity and activity as compared to peat. Accordingly, composts and management residues may have a positive effect on plant growth and resistance to pathogens. Additionally, several known biocontrol agents, such as *Bacillus* spp., *Pseudomonas* spp., and *Trichoderma* spp., have been retrieved from composts, which may contribute to a possible disease suppressive effect in composts (Dukare et al., 2011; Chen et al., 2012; Antoniou et al., 2017; Lutz et al., 2020). Other biocontrol agents associated with disease suppression in composts include non-pathogenic *Fusarium* spp. (Kavroulakis et al., 2007; Blaya et al., 2016), *Zopfiella* spp. (Blaya et al., 2016), *Enterobacter* spp. (Kwok et al., 1987; Chen et al., 2012), *Xanthomonas* spp. (Kwok et al., 1987), *Aeromonas* spp. (Oberhänsli et al., 2017), *Flavobacterium* spp. (Kwok et al., 1987) and non-pathogenic *Verticllium* spp. (Postma et al., 2003). In addition to biocontrol agents naturally occurring in composts, composts have been shown to improve colonization and consequently the efficacy of commercial biocontrol organisms (Krause et al., 2001; Joos et al., 2020).

Another important requirement for the use of peat alternatives in horticultural substrates is the absence of human and plant pathogens, as this may pose a potential risk for plant and human health (Jones and Martin, 2003). Several studies have shown the presence of pathogenic fungi that can infect plants *via* the roots, such as *Fusarium* spp., *Rhizoctonia* spp., and *Pythium* spp., in horticultural substrates (Cartwright, 1995; Waller et al., 2008). Potential human pathogens that have been reported to be present in substrates include *Salmonella* spp., *Escherichia* spp., *Shigella* spp., and *Klebsiella* spp. (Epstein, 2001; Jones and Martin, 2003).

A range of different feedstocks and processing methods make composts and management residues very heterogenous materials. Microbiological characteristics are also expected to show a large heterogeneity. Pot et al. (2021a,b) showed that the initial microbiological composition is paramount in obtaining a favorable microbiome in substrates, as possibilities for adaptation or optimization of microbiological characteristics of composts and management residues are limited. Hence, it is important to understand what properties drive the microbial composition of peat alternatives. Feedstock, pH, mineral N content and organic matter content have been suggested as potential drivers of microbial communities in composts (Neher et al., 2013; Sun et al., 2020; Wu et al., 2020). It is, however, unclear which properties drive the microbial composition in other types of composts and other peat alternatives.

The objective of this study is to compare microbiological characteristics of subtypes of composts and management residues to peat-based substrates using a classification based on feedstock that is also used by commercial suppliers. Specifically, this study focusses on how the microbiological characteristics of feedstock-based subtypes of composts and management residues differ from peat-based substrates, and how feedstock and (bio)chemical characteristics drive these microbiological characteristics. Moreover, this study assesses if these different subtypes of composts and management residues can be regarded as good peat alternatives based on different characteristics that may indicate plant growth and health promotion. These characteristics include presence of genera associated with known beneficial microorganisms, absence of genera known to include pathogens, high microbial diversity, high functional diversity and activity, high microbial biomass, high fungal to bacterial ratio and the potential to increase the inoculation efficiency of the biocontrol fungus *Trichoderma harzianum.* Finally, it was determined which microbiological characteristics may predict inoculation efficiency.

## Materials and methods

### Set of materials

The set of materials consisted of 10 peat-based substrates, 16 composts from different installations and feedstocks, and 12 management residues from various locations and vegetation types (Table 1). Composts and management residues were each divided into subtypes based on feedstock, as is common practice in the sector. For composts, four feedstock-based subtypes could be distinguished: green composts (C1), vegetable, fruit and garden (VFG) composts (C2), woody composts (C3), and peat composts (i.e., composts based on spent substrates; C4). For management residues, four feedstock-based subtypes could be distinguished: grass clippings (M1), chopped heath (M2), forest sods (M3) and woody fractions of composts (M4). Peat-based substrates were divided into two subtypes based on whether they were treated with lime. The two subtypes were classified as pure peat-based substrates (P1) and limed peat-based substrates (P2). An overview of (bio)chemical characteristics (determined and described by Vandecasteele et al. (2021)) of the different samples can also be found in Table 1.

**Table 1:** (Bio)chemical characteristics of the different samples of composts (C), management residues (M) and peat-based substrates (P).

Sample	Description	Type	Subtype	Cellulose (%/OM)	Hemicellulose (%/OM)	Lignin (%/OM)	pH-H_2_O	EC	NO_3_-N (mg/L)	NH_4_-N (mg/L)	N_min_ (mg/L)	SO_4_ (mg/L)	Cl (mg/L)	OM (%/DM)	P_water_ (mg/L)	C_water_ (mg/L)	C/N	N_immob_ (%)	OUR (mmol O_2_/kg OM/h)	Cum. CO_2_ release (mol CO_2_/kg OM)
BW01	Grass clippings	M	M1	16.3	10.1	15.6	5.6	39.0	<5.0	<5.0	<5.0	<11.7	11.1	50.7	<4.7	77.5	27.5	−11.0	8.3	0.8
BW02	Chopped heath	M	M2	27.5	17.9	27.0	5.6	24.0	<5.0	<5.0	<5.0	<11.7	<10	86.7	<4.7	47.7	32.3	3.0	7.7	1.4
BW03	Grass clippings	M	M1	33.4	38.2	8.6	7.1	189.0	<5.0	21.4	21.4	31.6	92.8	94.5	11.4	225.2	23.6	−8.0	24.6	2.4
BW04	Grass clippings	M	M1	7.9	8.4	7.6	4.8	198.0	63.9	5.4	69.3	<11.7	27.9	31.7	33.3	58.1	17.7	−7.0	4.2	1.4
BW05	Forest sods	M	M3	17.6	9.1	31.4	4.3	28.0	<5.0	<5.0	<5.0	<11.7	<10	77.9	<4.7	36.1	22.4	−2.0	2.2	0.6
BW06 #	Green compost	C	C1	8.8	3.3	7.8	8.8	851.0	6.3	<5.0	6.3	247.0	529.6	25.5	13.0	220.5	13.0	4.0	6.4	1.3
BW08 #	Green compost	C	C1	12.1	4.2	12.6	8.5	1156.0	<5.0	14.0	14.0	378.5	807.3	43.3	21.2	393.8	15.9	6.0	11.7	3.5
BW09 #	Wood chip compost	C	C3	9.6	7.8	11.8	7.6	2430.0	668.2	16.3	684.5	820.5	483.1	54.6	38.8	298.7	13.9	−5.0	1.6	0.3
BW10 #	Wood chip compost	C	C3	9.4	5.4	15.2	5.8	1209.0	367.9	40.8	408.7	400.6	175.0	43.3	78.1	129.9	15.9	−1.0	1.8	0.6
BW11 #	Peat compost	C	C4	13.2	9.4	16.0	6.4	2100.0	586.9	26.8	613.7	990.8	266.6	65.0	110.5	222.8	18.0	13.0	1.1	0.3
BW12 #	Peat compost	C	C4	28.1	10.7	31.7	6.9	1253.0	401.0	<5.0	401.0	1041.4	165.6	82.3	12.9	38.0	24.0	10.0	1.2	0.5
BW13 #	Poplar bark compost	C	C3	30.3	11.9	29.4	5.4	461.0	<5.0	<5.0	<5.0	28.1	66.2	88.4	42.0	352.7	46.5	63.0	7.8	4.8
BW14 #	Fungus-dominant woody compost	C	C3	8.1	5.2	11.3	7.5	361.0	<5.0	<5.0	<5.0	201.8	180.8	37.2	<4.7	87.3	16.8	21.0	2.8	1.6
BW15	Woody fraction of green compost	M	M4	46.8	19.3	23.3	6.9	234.0	<5.0	<5.0	<5.0	32.6	76.1	95.2	28.3	174.8	72.1	45.0	5.6	3.1
BW16 #	Green compost	C	C1	5.4	2.8	11.6	7.9	1273.0	193.0	10.9	203.9	96.9	640.9	30.9	28.9	274.5	11.1	−3.0	3.1	1.0
BW19 #	VFG compost	C	C2	7.1	5.4	11.1	8.5	3030.0	418.6	649.9	1068.5	1073.4	975.2	38.1	19.8	485.9	9.7	36.0	7.2	1.9
BW22	Woody fraction of green compost	M	M4	32.5	12.5	24.9	7.8	918.0	<5.0	15.3	15.3	173.7	710.8	79.8	29.9	379.8	37.6	38.0	11.1	3.7
BW24	Soft rush	M	M1	29.5	32.7	13.3	7.9	448.0	<5.0	30.4	30.4	156.0	266.2	89.2	23.6	159.9	21.7	7.0	15.7	3.5
BW25	Chopped heath	M	M2	16.7	11.7	17.2	6.0	31.0	<5.0	<5.0	<5.0	<11.7	<10	52.1	<4.7	92.7	30.8	78.0	12.0	2.6
BW26	Chopped heath	M	M2	5.5	4.0	9.7	4.5	333.0	88.4	101.4	189.8	79.5	17.5	36.2	13.9	71.3	16.8	−4.0	0.3	0.2
BW27	VFG compost	C	C2	7.8	3.2	11.6	8.5	2200.0	241.7	122.3	364.0	166.5	1663.9	32.9	27.9	308.9	8.6	−58.3	2.4	1.1
BW28	Woody fraction of green compost	C	C1	13.8	4.8	16.0	7.5	804.0	<5.0	8.4	8.4	168.1	363.7	48.1	23.3	563.7	15.9	44.0	16.0	5.6
BW29	Green compost	C	C1	12.1	4.9	17.4	8.1	894.0	77.2	7.7	84.9	108.5	477.5	50.0	34.2	265.3	12.1	−38.0	2.2	1.2
BW30	VFG compost mixed with green compost	C	C2	9.1	5.8	14.9	8.3	1490.0	170.7	76.5	247.2	401.7	986.1	44.8	31.5	242.3	9.8	−34.0	5.4	1.2
BW31	Green compost	C	C1	8.7	6.1	14.3	8.3	1721.0	225.1	6.1	231.2	330.1	1075.2	39.0	29.6	272.6	10.2	−27.9	3.0	1.3
BW32	Green compost	C	C1	10.3	4.4	11.9	8.9	1558.0	7.7	225.5	233.2	223.0	1276.2	41.0	56.0	415.7	10.8	43.7	4.8	2.2
BW34	Forest sods	M	M3	14.1	7.1	14.2	4.7	100.0	<5.0	<5.0	<5.0	<11.7	37.6	41.1	<4.7	89.0	27.0	−6.0	5.7	1.1
BW53	Chopped heath	M	M2	8.6	5.3	12.9	5.9	226.0	24.3	6.8	31.1	42.2	106.5	36.8	69.1	98.3	20.1	52.7	6.1	1.1
BWr61 *	White peat	P	P1	49.7	23.3	13.1	4.7	26.0	<5.0	<5.0	<5.0	<11.7	<10	97.8	<4.7	49.5	68.2	3.8	0.5	0.2
BWr62 *	Peat mixture	P	P2	31.0	18.3	24.5	6.1	231.0	50.6	<5.0	50.6	159.8	25.9	89.9	55.5	96.4	44.6		0.9	0.2
BWr67 *	Black peat	P	P1	21.6	9.1	32.1	4.7	272.0	17.5	<5.0	17.5	468.8	27.3	89.0	<4.7	31.5	37.7		1.2	0.1
BWr65	Peat mixture	P	P1	−160.0	17.5	21.5	4.0	164.0	20.7	34.8	55.5	173.7	21.5	97.6	<4.7	32.9	55.0	21.3	0.7	0.1
BWr66	Black peat	P	P1	28.6	17.3	31.2	4.7	33.0	<5.0	5.0	<5.0	27.8	22.5	95.9	4.7	60.4	36.9	7.0	0.7	0.3
BWr46	Peat mixture	P	P2	26.0	14.3	18.1	6.6	68.0	8.9	<5.0	8.9	63.3	23.7	69.1	<4.7	51.9	38.7		0.3	
BWr47	Peat mixture	P	P2	26.2	9.2	21.7	6.0	702.0	275.8	<5.0	275.8	486.4	26.4	69.5	78.2	26.4	42.0		0.1	0.2
BWr48	Peat mixture	P	P2	16.3	7.2	11.5	6.4	86.0	8.6	<5.0	8.6	80.7	26.5	33.7	17.5	53.1	28.3		0.6	
BWr49	Peat mixture	P	P2	28.2	10.0	31.6	6.7	128.0	10.6	<5.0	10.6	159.8	23.8	80.6	<4.7	41.8	47.5		0.3	
BWr419	White peat	P	P1	−124.1	14.3	19.2	4.9	129.0	<5.0	5.7	5.7	<11.7	<10	97.2	<4.7	52.8	47.3	20.6	0.2	0.3

P1 = pure peat-based substrates (*n* = 5); P2 = limed peat-based substrates (*n* = 5); C1 = green composts (*n* = 7); C2 = VFG composts (*n* = 3); C3 = woody composts (*n* = 4); C4 = peat composts (*n* = 2); M1 = grass clippings (*n* = 4); M2 = chopped heath (*n* = 4); M3 = forest sods (*n* = 2); M4 = woody fractions of composts (*n* = 2). Asterisks indicate reference samples used for community-level physiological profiling (Biolog EcoPlates). Hashtags indicate compost samples used for inoculation efficiency. VFG compost, vegetable, fruit and garden compost; EC, electrical conductivity; N_min_, mineral N = NO_3_–N + NH_4_–N; OM, organic matter; DM, dry matter; P_water_, water-extractable P; C_water_, water-extractable C; N_immob_, N immobilization; OUR, oxygen uptake rate.

### 16S rRNA and ITS2 gene metabarcoding

The different materials were each sampled three times (250 mg per sample), resulting in three technical replicates for each sample. DNA was extracted from each sample using the DNeasy Powersoil Pro Kit (QIAGEN, Germantown, MD, United States), according to the manufacturer’s instructions, and stored at −20°C until use for metabarcoding, as described below.

Metabarcoding of the bacterial and fungal populations was done on the V3-V4 fragment of the 16S rRNA gene and the ITS2 gene fragment, respectively, as described in detail in De Tender et al. (2016a). Reads are available for download at the NCBI sequence read archive (SRA) under project numbers PRJNA624053, PRJNA715731 and PRJNA767265.

Demultiplexing of the metabarcoding dataset was performed by the sequencing provider. Primers were removed using Trimmomatic version 0.32 (Bolger et al., 2014). Adapters were already removed by the sequencing provider. For the ITS2-sequences, some adapters were still present and were removed using Cutadapt version 2.7 (Martin, 2011). Quality of the pre-processed sequences was checked using FastQC version 0.11.8 (Andrews, 2010). Further processing of the sequences was done using the DADA2 pipeline version 1.12.1 (Callahan et al., 2015), as described in detail in Pot et al. (2021a). Briefly, low quality reads were trimmed, sequences were dereplicated and amplicon sequence variants (ASVs) were inferred based on the parametric model of errors calculated by the algorithm. Inferred sequences were merged, chimeras were removed and taxonomy was assigned by the SILVA database v132 (bacteria; Quast et al., 2012; Yilmaz et al., 2013; Glöckner et al., 2017) and UNITE database v020219 (fungi; Nilsson et al., 2018).

Two sequence tables (bacterial and fungal) were constructed. For each biological replicate (*n* = 10 for peat-based substrates, *n* = 16 for composts and *n* = 12 for management residues), the mean of the absolute ASV counts of the tree technical replicates was calculated. All analyses were done for both the bacterial and fungal sequence tables. To remove low abundant reads, first, ASVs with less than three counts per million in at least three samples were removed from the datasets. Second, the table was used as input to calculate the Shannon diversity index applying the diversity function of the vegan package (version 2.5.7) in R (version 4.0.4; Oksanen et al., 2020), to determine alpha diversity. To find significant differences in mean diversity between the different subtypes of composts, management residues and peat-based substrates, a linear model including subtype as main effect was used. Linearity, homogeneity of variances and normality were checked prior to analysis by plotting residuals vs. fitted values, a QQ plot of the standardized residuals and a scale-location plot. Pairwise comparisons were made using least square means. *p*-Values <0.05 were considered significant. Third, beta diversity was studied. Absolute ASV counts were transformed to relative abundances, and a dissimilarity matrix (based on the Bray–Curtis dissimilarity index) was calculated from the ASV table. Homogeneity of the variances was checked on this dissimilarity matrix using the betadisper function. The effect of type of biomass and subtype on the community composition was studied by doing a PERMANOVA analysis on the dissimilarity matrix. To visualize the observed differences, principal coordinate analysis (PCoA) on the dissimilarity matrix was done. Fourth, heatmaps were made using the heatmap.2 function of the gplots package (version 3.1.1) in R for each type of biomass to visualize similarities between different samples. As input for these heatmaps, bacterial and fungal genera with a relative abundance equal to or larger than 1% in at least one of the samples were used. Fifth, (bio)chemical characteristics of the different samples, were fitted onto the PCoA ordinations for each type of biomass using the envfit function of the vegan package (version 2.5.7). More specifically, cellulose, hemicellulose, pH-H_2_O, electroconductivity (EC), nitrates (NO_3_-N), ammonium (NH_4_-N), mineral N (N_min_), sulfates (SO_4_), chlorine (Cl), organic matter (OM), water extractable phosphor (P_water_) and carbon (C_water_), carbon:nitrogen ratio (C/N), nitrogen immobilization (N_immob_), oxygen uptake rate (OUR) and cumulative CO_2_ release, that were determined and described in detail by Vandecasteele et al. (2021) and that can be found in Table 1, were used for this analysis. Significance of the correlations between the (bio)chemical characteristics and the PCoA ordination on the other hand was tested using a permutation test with 999 permutations. Significant correlations (*p* < 0.05) were plotted on the PCoA plots with the length of the arrows proportional to the correlation. Sixth, the presence of potential beneficial microorganisms was studied, focusing on genera known to include plant growth promoting microorganisms and biocontrol agents, including *Penicillium*, *Serratia*, *Paenibacillus*, *Burkholderia*, *Trichoderma*, *Bacillus*, *Pseudomonas*, and *Streptomyces* (Bhattacharyya and Jha, 2012; Neher et al., 2022). Additionally, the different samples were screened for the presence of genera including potential pathogens, focusing on genera known to include human pathogens, including *Salmonella*, *Escherichia*, *Klebsiella*, *Shigella*, and *Enterobacter*, or plant pathogens that can infect the plant roots *via* the growing medium, including *Verticillium*, *Rhizoctonia*, *Fusarium*, *Pythium*, *Sclerotinia*, and *Plasmodiaphora*. Seventh, the effect of subtype of biomass on abundance was tested using the edgeR package (version 3.32.1; Robinson et al., 2010) as described in Pot et al. (2021a). The analyses were done upon clustering the bacterial and fungal ASV table with absolute sample counts at phylum, family, and genus level. Normalization based on the trimmed mean of M-values (TMM) was applied to correct for differences in library size of the count table. A design matrix was defined based on the experimental design, with a main effect for subtype. The dispersion parameter was calculated. Following, a negative binomial model was fitted for every ASV and then combined. Likelihood-ratio tests were conducted on the contrast of the model parameters to assess differential abundances. *p*-Values <0.05 were considered significant. Correction for multiple testing was included by adopting the Benjamini-Hochberg False Discovery Rate procedure.

### Community-level physiological profiling using Biolog EcoPlates

The different materials were each sampled once (3 g per sample) and analyzed using Biolog EcoPlates (Biolog, Inc., CA, United States) as described in detail in Pot et al. (2021a). For peat-based substrates, only three samples were used (see Table 1). The average well color development (AWCD) and Shannon diversity index (functional diversity) were calculated as described in Pot et al. (2021a). For each biological replicate, the average AWCD and Shannon diversity index was calculated from the three technical replicates.

To determine differences in overall AWCD and AWCD of the different carbon sources and the functional diversity (Shannon diversity index) between the subtypes of biomass, a linear model including subtype as main effect was used after checking the assumptions. Pairwise comparisons were made using least square means. Furthermore, relative optical density values after 7 days were divided by the AWCD to minimize the influence of inoculum density differences between plates (Garland and Mills, 1991; Graham and Haynes, 2005). To visualize differences in functional community composition, principal component analysis (PCA) was done on these values. The effect of subtype of biomass was studied by doing a PERMANOVA analysis.

### Phospholipid fatty acid analysis

Phospholipid fatty acid (PLFA) analysis was performed by Vandecasteele et al. (2021). Seventeen PLFAs were selected because of their use of biomarker fatty acids for six distinct microbial groups: Gram-positive bacteria (i-C15:0, a-C15:0, i-C16:0, i-C17:0), Gram-negative bacteria (C16:1c9, C17:0cy, C19:0cy), bacteria (non-specific; C14:0, C15:0, C16:0, C17:0, C18:0), actinomycetes (10Me-C16:0, 10Me-C18:0), fungi (C18:2n9,12) and mycorrhiza (C16:1c11), and summed up together with C18:1c9 to calculate total microbial biomass. In addition, fungal to bacterial ratio was determined.

To determine differences in total microbial biomass and fungal to bacterial ratio between subtypes, a linear model was used with subtype as main effect after checking the assumptions. Pairwise comparisons were made using least square means. To visualize differences in microbial biomass between subtypes, principal component analysis (PCA) was done on the microbial biomass of different microbial groups and total microbial biomass. The effect of subtype of biomass was studied by doing a PERMANOVA analysis.

### Inoculation efficiency of *Trichoderma harzianum*

Inoculation efficiency by the biocontrol fungus *T. harzianum* was assessed by qPCR as described in Vandecasteele et al. (2021). Differences in inoculation efficiency between subtypes of composts, management residues and peat-based substrates were determined using a linear model with subtype as a main effect after checking the assumptions. Pairwise comparisons were made using least square means. Spearman correlations were used to determine correlations between the inoculation efficiency and the initial microbiological characteristics of the samples (bacterial and fungal diversity, biomass of different microbial groups, metabolic activity, and functional diversity).

All statistical tests were conducted in RStudio 1.2.5001.

## Results

### Comparison between peat-based substrates, composts and management residues

Differences in bacterial and fungal community composition between composts, management residues and peat-based substrates were visualized by principal coordinate analysis (PCoA; Supplementary Figure S1). For bacteria, the first and second principal coordinate (PCo) represented 12.9% and 8.1%, respectively, of the variance in the dataset, whereas for the fungal communities, these values were 10.8% and 9.8%, respectively. Particularly for bacteria, PCo1 represented variation between the different types of biomass (i.e., composts, management residues and peat-based substrates), while PCo2 represented variation between the individual samples within the three types of biomass. PERMANOVA analysis showed a significant shift in the bacterial communities (*p* = 0.001) and fungal communities (*p* = 0.001) between the types of biomass. Composts and management residues both show large variation in their bacterial and fungal community composition, indicating high heterogeneity within the microbial communities of each type of biomass.

### Comparison between subtypes of peat-based substrates and composts and management residues

#### Differences in microbial community composition

**Figure 1:**
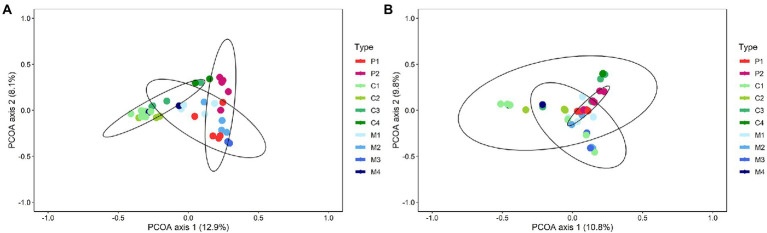
Shifts in bacterial **(A)** and fungal **(B)** community composition between the subtypes of peat-based substrates, composts, and management residues. Both figures represent Principal Coordinate Analysis (PCoA) profiles of pairwise community dissimilarity (Bray-Curtis) indices of either bacterial (16S V3-V4 rRNA gene) or fungal (ITS2 gene) sequencing data, respectively. Colors indicate the different subtypes of peat-based substrates, composts, and management residues. P1 = pure peat-based substrates (*n* = 5); P2 = limed peat-based substrates (*n* = 5); C1 = green composts (*n* = 7); C2 = VFG composts (*n* = 3); C3 = woody composts (*n* = 4); C4 = peat composts (*n* = 2); M1 = grass clippings (*n* = 4); M2 = chopped heath (*n* = 4); M3 = forest sods (*n* = 2); M4 = woody fractions of composts (*n* = 2).

Redoing the PCoA with the subtypes as input, differences in bacterial and fungal community composition were still observed between the subtypes of composts, management residues and peat-based substrates, for both bacteria and fungi (*p* = 0.001 and *p* = 0.001, respectively; Figure 1). However, the condition of homogeneity of variances was not fulfilled for fungi (*p* < 0.001), indicating that the division in subtypes might be not sufficient to deal with the high sample heterogeneity.

Next to the differences between peat-based substrates, composts and management residues, also within each type of biomass differences in bacterial and fungal community composition were found (Supplementary Figure S2). PERMANOVA analysis showed a significant difference in the bacterial and fungal community composition between the different subtypes within composts (*p* = 0.001 and *p* = 0.003, respectively) and peat-based substrates (*p* = 0.02 and *p* = 0.02, respectively). For management residues, a significant difference in bacterial community composition was found between the subtypes (*p* = 0.01).

To verify whether this heterogeneity in the community is indeed dependent on feedstock-based subtypes within each type of biomass, heatmaps were produced based on the genera with a relative abundance of at least 1% in one of the samples to visualize similarities between the different samples (Supplementary Figures S3, S4). For peat-based substrates, the two feedstock-based subtypes – pure and limed peat-based substrates – showed a similar clustering based on bacterial and fungal community composition. Only one sample of the pure peat-based substrates clustered more closely to the limed peat-based substrates than to the other pure peat-based substrates, which could also be noted in the PCoA plots. For composts, no real clustering on feedstock could be noted, either for the bacterial and fungal community. This is in contrast of what could be observed in the PCoA plots (Supplementary Figure S4): samples of the different feedstock-based subtypes clustered relatively closely together for both bacterial and fungal sequences, indicating samples belonging to feedstock-based subtypes show similar bacterial and fungal community composition. Green composts (C1) and VFG composts (C2) showed similar bacterial and fungal community compositions. Woody composts (C3) and peat composts (C4) also showed similar bacterial and fungal community compositions. For management residues, samples of the different feedstock-based subtypes showed less similarity in their bacterial and fungal community composition. Samples belonging to forest sods (M3) or woody fractions of composts (M4) each showed similar bacterial and fungal community composition. However, samples of grass clippings (M1) and samples of chopped heath (M2) showed large variation in bacterial and fungal community compositions, which could be noted in the bacterial and fungal heatmaps as well as in the PCoA plots. Except for the composts, the heatmaps and PCoA plots showed the same patterns. The differences between the PCoA plots and the heatmaps for composts may be due to differences in the determination of similarities between samples. In the PCoA plots, the total bacterial and fungal community composition is considered, while the heatmaps are based on genera that have a relative abundance of at least 1% in at least one sample.

#### Linking microbial community composition with chemical characteristics

**Figure 2:**
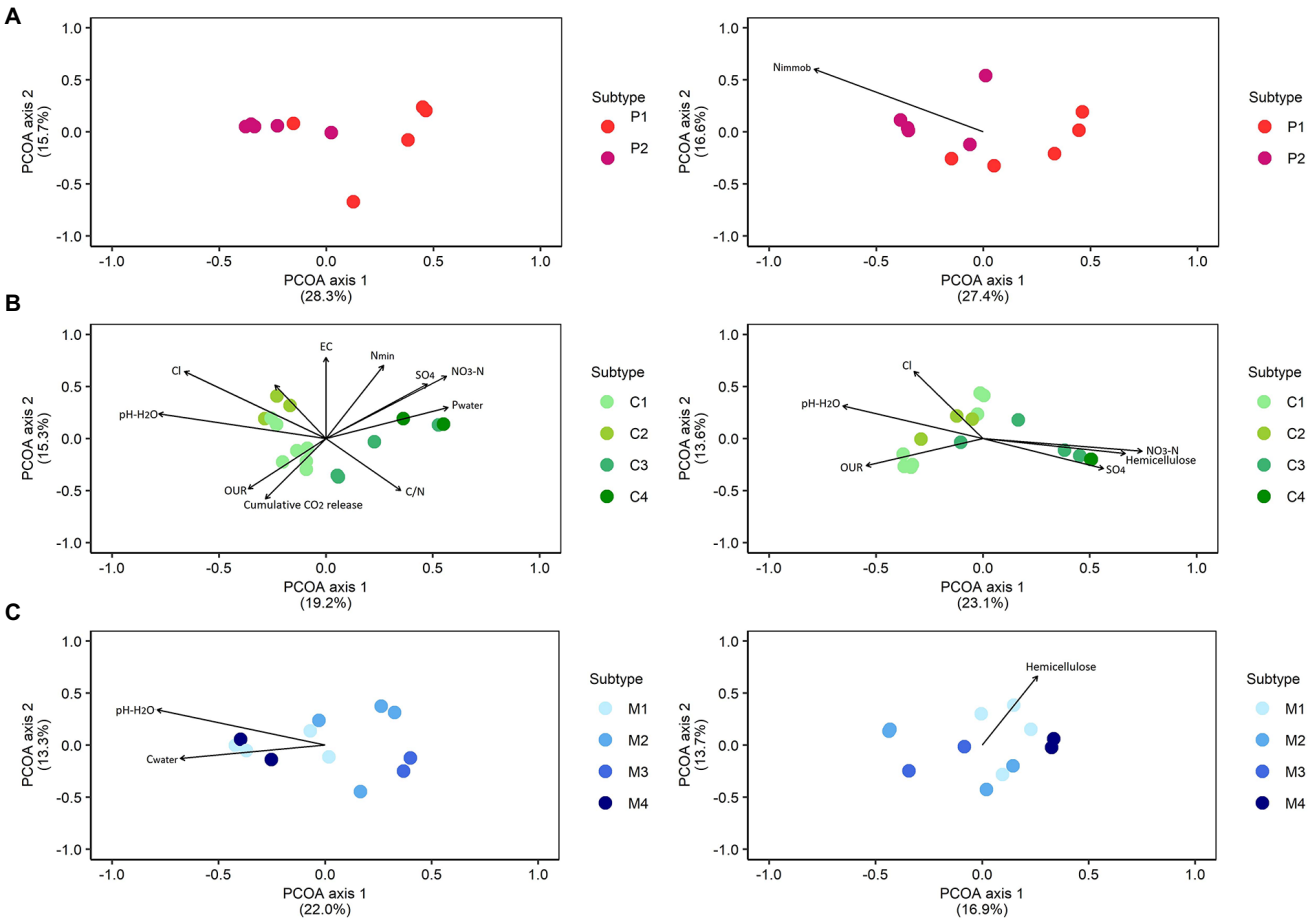
Correlations between bacterial and fungal community composition and chemical characteristics. Principal Coordinate Analysis (PCoA) profile of pairwise community dissimilarity (Bray-Curtis) indices of bacterial (16S V3-V4 rRNA gene; left) and fungal (ITS2 gene; right) sequencing data in peat-based substrates **(A)**, composts **(B)**, and management residues **(C)**. Arrows indicate significant correlations between corresponding variables and microbial community composition. The segments are scaled to the *r*^2^ value, so that variables with a longer segment are more strongly correlated with the data than those with a shorter segment. Nimmob = N immobilization; Pwater = water extractable P; Fungi = total biomass fungi; Actinomycetes = total biomass actinomycetes; Gram – bact. = total biomass Gram-negative bacteria; Gram + bact. = total biomass of Gram-positive bacteria; Non-specific bact. = total biomass of non-specific bacteria. P1 = pure peat-based substrates (*n* = 5); P2 = limed peat-based substrates (*n* = 5); C1 = green composts (n = 7); C2 = VFG composts (*n* = 3); C3 = woody composts (*n* = 4); C4 = peat composts (*n* = 2); M1 = grass clippings (*n* = 4); M2 = chopped heath (*n* = 4); M3 = forest sods (*n* = 2); M4 = woody fractions of composts (*n* = 2).

Within each type of biomass, the correlations between the bacterial and fungal community composition and chemical characteristics were determined (Figure 2; Supplementary Table S1). For peat-based substrates, no (bio)chemical characteristics were significantly correlated with the bacterial community composition. Fungal community composition in peat-based substrates was significantly correlated with N immobilization (*p* = 0.04, *r*^2^ = 0.99). For composts, bacterial community composition was significantly correlated with pH-H_2_O (*p* = 0.003), EC (*p* = 0.004), NO_3_-N (*p* = 0.002), NH_4_-N (*p* = 0.05), N_min_ (*p* = 0.004), SO_4_ (*p* = 0.02), Cl (*p* = 0.001), P_water_ (*p* = 0.03), C/N ratio (*p* = 0.03), oxygen uptake rate (OUR; *p* = 0.05) and cumulative CO_2_ release (*p* = 0.04), for which Cl had the highest influence on the bacterial community composition in composts (*r*^2^ = 0.85). Fungal community composition in composts was significantly correlated with hemicellulose content (*p* = 0.02), pH-H_2_O (*p* = 0.008), NO_3_-N (*p* = 0.006), SO_4_ (*p* = 0.05), Cl (*p* = 0.009) and oxygen uptake rate (OUR; *p* = 0.05), for which NO_3_-N had the highest influence on the bacterial community composition in composts (*r*^2^ = 0.57). For management residues, the bacterial community composition was significantly correlated with pH-H_2_O (*p* = 0.006, *r*^2^ = 0.74) and C_water_ (*p* = 0.04, *r*^2^ = 0.48), and the fungal community composition was solely correlated with hemicellulose (*p* = 0.02, *r*^2^ = 0.51).

#### Differences in characteristics of the microbial community

To study the difference in microbial community between the subtypes of the three biomass types in more detail, (1) differential abundances between peat-based substrates and management residues/composts, (2) the presence of beneficial microorganisms and pathogens, and (3) bacterial and fungal diversity were investigated.

First, the differential abundances of bacterial and fungal phyla, families, and genera between the subtypes of composts and management residues on one hand and peat-based substrates on the other hand were studied (Supplementary Tables S2, S3). For bacteria, the number of differentially abundant taxa in subtypes of composts and management residues was larger when compared to limed peat-based substrates than to pure peat-based substrates, indicating that bacterial community composition in subtypes of composts and management residues is more similar to pure peat-based substrates. For fungi, the number of differentially abundant taxa in subtypes of composts and management residues was similar when compared to either pure or limed peat-based substrates, indicating that subtypes of composts and management residues show a similar level of (dis)similarity as compared to pure or limed peat-based substrates. The relative number of significantly differential abundant taxa in compost and management residues is considerable smaller for fungi than for bacteria, indicating the fungal community composition of composts and management residues is more similar to that of peat-based substrates than the bacterial community composition. For composts, green composts (C1) showed the largest number of differentially abundant bacterial genera as compared to pure (P1; 76 genera) and limed peat-based substrates (P2; 268 genera), while woody composts (C3) showed the largest number of differentially abundant fungal genera as compared to pure (P1; 7 genera) and limed peat-based substrates (P2; 6 genera). For management residues, woody fractions of composts (M4) showed the largest number of differentially abundant bacterial genera as compared to pure (P1; 26 genera) and limed peat-based substrates (P2; 129 genera). Grass clippings (M1) showed the largest number of differentially abundant fungal genera as compared to pure peat-based substrates (P1; 8 genera), while chopped heath (M2) showed the largest number of differentially abundant fungal genera compared to limed peat-based substrates (P2; 14 genera).

A detail of the differentially abundant bacterial genera (relative abundance >1%) and fungal genera between subtypes of peat-based substrates P1 and P2 on one hand and subtypes of composts and management residues on the other hand is shown in Supplementary Tables S4–S7. There were no bacterial genera that were significantly increased or decreased compared to pure peat-based substrates (P1) in all subtypes of composts. All subtypes of composts showed a significant increase in the relative abundances of *Flavobacterium* as compared to limed peat-based substrates (P2). No fungal genera were significantly increased or decreased compared to pure (P1) or limed (P2) peat-based substrates in all subtypes of composts. No bacterial or fungal genera were significantly increased or decreased compared to pure (P1) or limed (P2) peat-based substrates in all subtypes of management residues.

**Figure 3:**
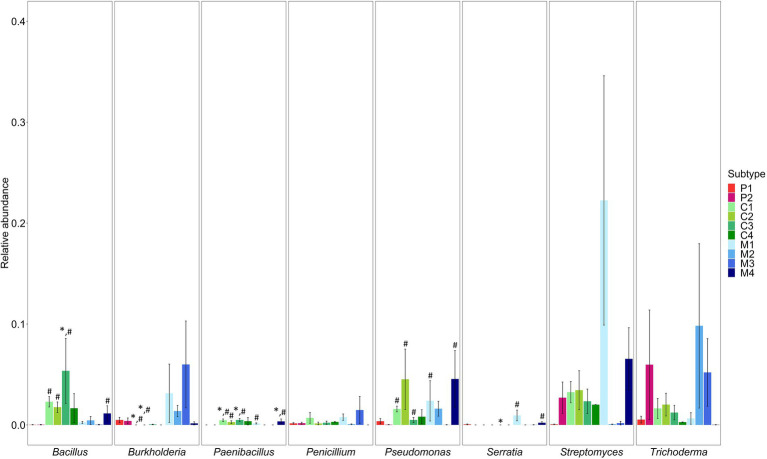
Mean relative abundances (proportions) ± SE of genera known to include beneficial microorganisms *Bacillus*, *Burkholderia*, *Paenibacillus*, *Pseudomonas*, *Serratia, Streptomyces*, *Penicillium* and *Trichoderma* in the different subtypes of peat-based substrates, composts, and management residues. Colors represent the different subtypes in each type of biomass. P1 = pure peat-based substrates (*n* = 5); P2 = limed peat-based substrates (*n* = 5); C1 = green composts (*n* = 7); C2 = VFG composts (*n* = 3); C3 = woody composts (*n* = 4); C4 = peat composts (*n* = 2); M1 = grass clippings (*n* = 4); M2 = chopped heath (*n* = 4); M3 = forest sods (*n* = 2); M4 = woody fractions of composts (*n* = 2). Asterisk indicates a significant difference as compared to P1. Hashtag indicates a significant difference as compared to P2.

Second, the presence of genera known to include beneficial microorganisms and genera known to include human and/or plant pathogens was determined. The genera associated with the potential beneficial microorganisms *Bacillus*, *Paenibacillus*, *Pseudomonas* and *Serratia* were differentially abundant in several subtypes of composts and management residues as compared to the subtypes of peat-based substrates (Supplementary Table S8; Figure 3). The relative abundance of *Bacillus* was significantly higher in woody composts (C3) than in pure peat-based substrates (P1; *p* < 0.001) and in green composts (C1; *p* = 0.003), VFG composts (C2; *p* = 0.006), woody composts (C3; *p* < 0.001) and woody fractions of composts (M4; *p* = 0.003) than in limed peat-based substrates (P2). *Paenibacillus* was significantly more abundant in green composts (C1; *p* < 0.001), woody composts (C3; *p* < 0.001) and woody fractions of composts (M4; *p* < 0.001) than in pure peat-based substrates (P1) and in green composts (C1; *p* < 0.001), VFG composts (C2; *p* < 0.001), woody composts (C3; *p* < 0.001), grass clippings (M1; *p* = 0.001) and woody fractions of composts (M4; *p* < 0.001) than in limed peat-based substrates (P2). *Pseudomonas* was significantly more abundant in green composts (C1; *p* = 0.006), VFG composts (C2; *p* = 0.002), woody composts (C3; *p* = 0.01), grass clippings (M1; *p* = 0.005) and woody fractions of composts (M4; *p* = 0.002) than in limed peat-based substrates (P2). The relative abundance of *Serratia* was significantly lower in woody composts (C3) than in pure peat-based substrates (P1; *p* < 0.001) and significantly higher in grass clippings (M1; *p* < 0.001) and woody fractions of composts (M4; *p* < 0.001) than in limed peat-based substrates (P2). *Burkholderia* was significantly less abundant in green composts (C1) and VFG composts (C2) than in pure (P1; *p* < 0.001 and *p* = 0.005, respectively) and limed peat-based substrates (P2; *p* < 0.001 and *p* = 0.005, respectively). *Streptomyces*, *Penicillium* and *Trichoderma* were not differential abundant in the subtypes of composts and management residues compared to the subtypes of peat-based substrates. Other genera that were significantly more abundant in at least one subtype of composts or management residues also have been found in literature to include beneficial species. Supplementary Table S9 shows an overview of these genera and the species that have been found to have a positive effect on disease suppression of plant pathogens in horticulture or to have plant growth promoting characteristics in horticultural plants. Most of these genera were significantly more abundant in green composts (C1), VFG composts (C2) and woody composts (C3) and in grass clippings (M1) and woody fractions of composts (M4). A larger number of these genera was significantly more abundant in the subtypes of composts and management residues when compared to limed peat-based substrates (P2) than compared to pure peat-based substrates (P1; see Supplementary Tables S4–S7).

Genera known to include potential human and/or plant pathogens *Klebsiella*, *Enterobacter* and *Escherichia/Shigella* were differentially abundant in several subtypes of composts and management residues as compared to the subtypes of peat-based substrates (Supplementary Table S10). *Klebsiella* was significantly more abundant in grass clippings (M1; *p* = 0.005) and woody fractions of composts (M4; *p* = 0.009) than in limed peat-based substrates. The relative abundance of *Enterobacter* was significantly higher in grass clippings (M1; *p* < 0.001 and *p* < 0.001) than in the two subtypes of peat-based substrates. Moreover, *Enterobacter* was significantly more abundant in green composts (C1; *p* = 0.02) and woody fractions of composts (M4; *p* < 0.001) than in limed peat-based substrates. The relative abundance of *Escherichia/Shigella* was significantly higher in grass clippings (M1; *p* = 0.001 and *p* = 0.001, respectively) and woody fractions of composts (M4; *p* = 0.006 and *p* = 0.006, respectively) than in pure (P1) and limed peat-based substrates (P2).

**Figure 4:**
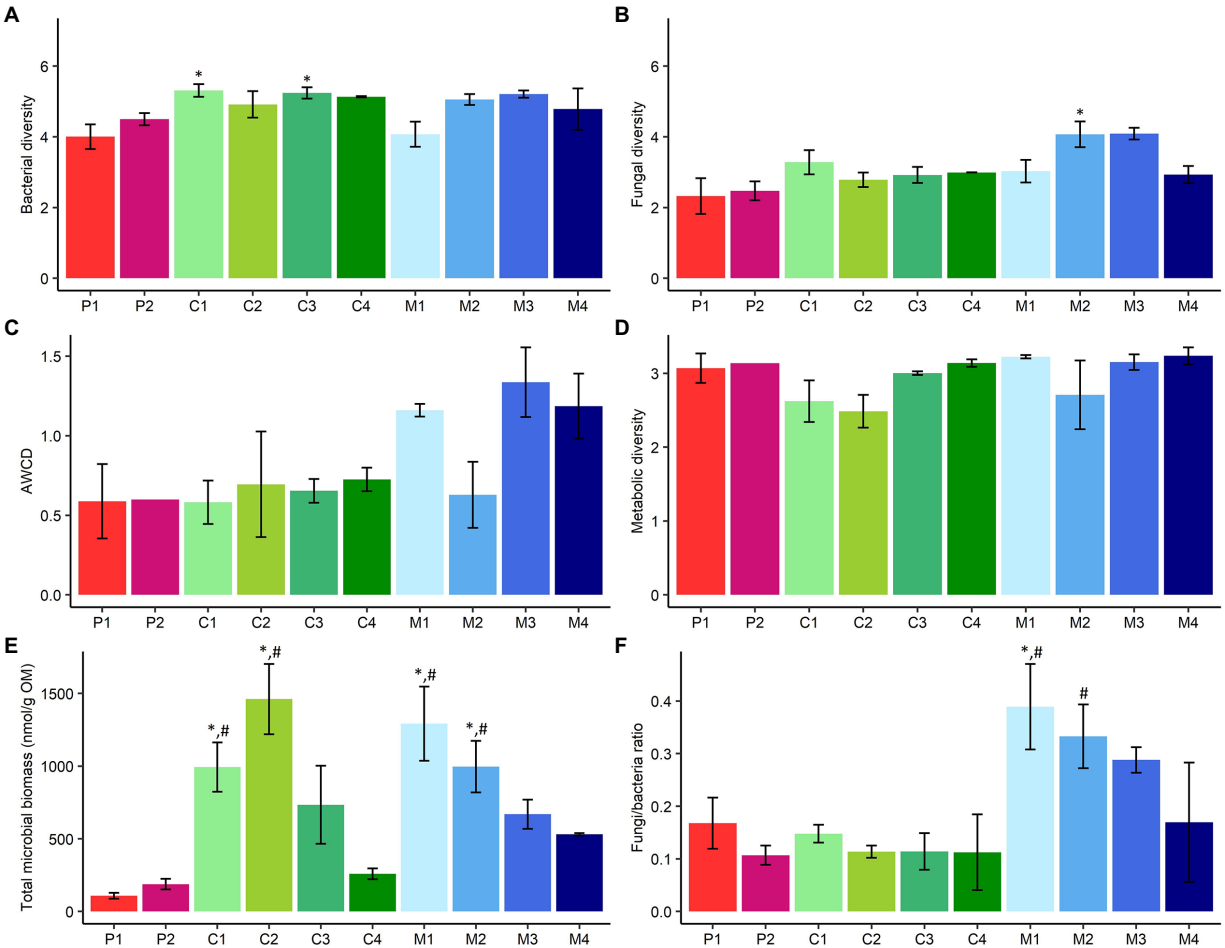
Comparison of microbiological characteristics of subtypes of peat-based substrates, composts, and management residues. **(A)** Mean bacterial diversity ± SE, calculated as the Shannon Diversity Index, based on 16S V3-V4 rRNA gene metabarcoding data. **(B)** Mean fungal diversity ± SE, calculated as the Shannon Diversity Index, based on ITS2 gene metabarcoding data. **(C)** Mean metabolic activity ± SE, expressed as AWCD (average well color development), based on data from community-level physiological profiling using Biolog EcoPlates. **(D)** Mean metabolic diversity ± SE, calculated as the Shannon diversity index, based on data from community-level physiological profiling using Biolog EcoPlates. **(E)** Mean total microbial biomass ± SE (nmol/g OM), based on PLFA analysis data. **(F)** Mean fungal to bacterial ratio (F/B) ± SE, based on PLFA analysis data. P1 = pure peat-based substrates (*n* = 5); P2 = limed peat-based substrates (*n* = 5); C1 = green composts (*n* = 7); C2 = VFG composts (*n* = 3); C3 = woody composts (*n* = 4); C4 = peat composts (*n* = 2); M1 = grass clippings (*n* = 4); M2 = chopped heath (*n* = 4); M3 = forest sods (*n* = 2); M4 = woody fractions of composts (*n* = 2). Asterisk indicates a significant difference as compared to P1, and hashtag indicates a significant difference as compared to P2.

Third, bacterial and fungal diversity in the different subtypes of composts, management residues and peat-based substrates were determined (Figures 4A,B). Green composts (C1; *p* = 0.05) and woody composts (C3; *p* = 0.04) showed a significant higher bacterial diversity than pure peat-based substrates (P1). Fungal diversity was significantly higher in chopped heath (M2; *p* = 0.04) than in pure peat-based substrates (P1).

#### Differences in functional characteristics of microbial community

Functional community composition was not significantly different between the different subtypes of biomass (Supplementary Figure S5). Metabolic activity, expressed as AWCD, showed no significant differences between the subtypes of peat-based substrates and subtypes of composts and management residues (Figure 4C). Metabolic diversity did not significantly differ between the subtypes of peat-based substrates and subtypes of composts and management residues (Figure 4D). No significant differences in AWCD of different C-sources were found between subtypes of composts and management residues and subtypes of peat-based substrates (Supplementary Figure S6).

#### Differences in microbial biomass

PCA showed a significant difference in microbial biomass between the different subtypes of biomass (Supplementary Figure S7). Green composts (C1; *p* = 0.009 and *p* = 0.02, respectively), VFG composts (C2; *p* < 0.001 and *p* = 0.001, respectively) have a significant higher microbial biomass than pure (P1) and limed peat-based substrates (P2). Grass clippings (M1; *p* < 0.001 and *p* = 0.002, respectively) and chopped heath (M2; *p* = 0.02 and *p* = 0.05, respectively) have a significant higher microbial biomass than pure (P1) and limed peat-based substrates (P2; Figure 4E).

Grass clippings (M1; *p* = 0.04 and *p* = 0.004, respectively) have a significant higher fungi/bacteria ratio than pure (P1) and limed peat-based substrates (P2). Chopped heath (M2) has a significant higher fungi/bacteria ratio than limed peat-based substrates (P2; *p* = 0.04; Figure 4F).

#### Inoculation with *Trichoderma harzianum*

No significant differences in inoculation efficiency were found between the different subtypes of composts and management residues (Supplementary Figure S8).

**Figure 5:**
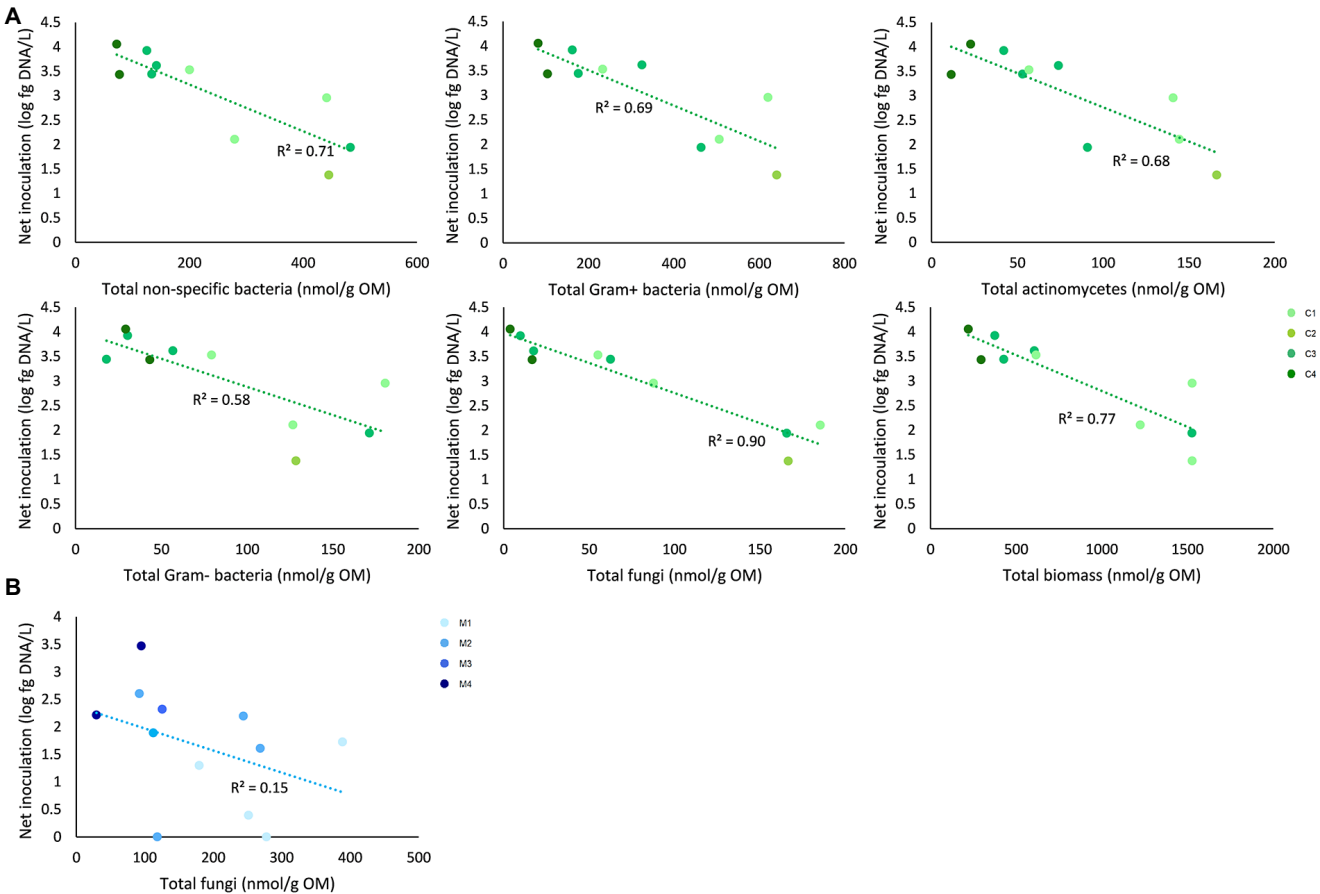
Correlations between initial microbial characteristics of composts and management residues and net inoculation of *Trichoderma harzianum*. **(A)** Net inoculation in composts was significantly correlated with the initial biomass of non-specific bacteria, Gram-positive bacteria, Actinomycetes, Gram-negative bacteria, fungi, and the total initial microbial biomass. **(B)** Net inoculation in management residues was significantly correlated with biomass of fungi. Determination coefficients (*R*^2^) are shown. Colors indicate the different subtypes of composts and management residues. C1 = green composts (*n* = 3); C2 = VFG composts (*n* = 1); C3 = woody composts (*n* = 4); C4 = peat composts (*n* = 2); M1 = grass clippings (*n* = 4); M2 = chopped heath (*n* = 4); M3 = forest sods (*n* = 2); M4 = woody fractions of composts (*n* = 2).

For peat-based substrates, net inoculation was not significantly correlated with the initial microbial characteristics. Net inoculation in composts was significantly correlated with the initial biomass of non-specific bacteria (*p* = 0.01; rho = −0.82), Gram-positive bacteria (*p* = 0.01; rho = −0.78), Actinomycetes (*p* = 0.02; rho = −0.73), Gram-negative bacteria (*p* = 0.02; rho = −0.73), fungi (*p* = 0.001; rho = −0.89), and the total initial microbial biomass (*p* = 0.01; rho = −0.77). In management residues, net inoculation was significantly correlated with the initial biomass of fungi (*p* = 0.04; rho = −061; Figure 5).

## Discussion

Composts, management residues and peat-based substrates showed differences in their microbial community composition. However, even within each type of biomass, a high in-between sample variability in the bacterial and fungal community could be noted. To look deeper into this variability, the three types of biomass were classified in subtypes using a feedstock-based classification that is also used by commercial suppliers. Based on these subtypes, the microbiological characteristics of composts, management residues were studied in comparison to peat-based substrates in three ways.

First, it was assessed how feedstock and (bio)chemical characteristics drive microbiological characteristics of subtypes of peat-based substrates, composts, and management residues and how these subtypes of compost and management residues differ from peat-based substrates.

Samples of pure peat-based substrates showed a different microbial community than limed peat-based substrates, indicating that liming of the substrates influences the microbiome. Therefore, for microbiological characteristics, the classification based on liming of the peat-based substrates seems relevant. Bacterial community composition was not related to any other (bio)chemical characteristics. Differences in fungal community composition were related to nitrogen immobilization. Jezile et al. (2009) showed that the addition of lime to soil can cause an increase in nitrogen immobilization caused by a higher microbial activity, which may explain differences in nitrogen immobilization between pure and limed peat-based substrates. Differences in N immobilization in soils can also be linked to differences in microbial community composition (Schimel et al., 2005).

For composts, samples of feedstock-based subtypes clustered relatively closely together, indicating a similar microbiological composition within each feedstock-based subtype. For other types of compost, Ashraf et al. (2007) and Neher et al. (2013) also showed that bacterial and fungal communities responded to feedstock, resulting in distinct types of microbial communities in composts produced from different materials. In this study, there was, however, considerable overlap between several feedstock-based subtypes of composts. Microbial community composition of green composts and VFG composts showed large overlap, especially for fungal community composition. These similarities in microbial community composition may be due to similarities in feedstock, as there is a large diversity of source materials for both subtypes. Reyes-Torres et al. (2018) showed high variability in the composition of green composts due to the diversity of source materials. Woody composts and peat composts also showed large overlap, for both bacterial and fungal community composition. Again, similarities in feedstock may cause the similarities in microbial community composition for both subtypes of compost. Differences in bacterial community composition between different composts were most strongly related to chlorine content and nitrate, while differences in fungal community composition were most strongly related to nitrate. Other studies also reported that bacterial and fungal community composition were affected by nitrate (Zhang et al., 2011; Wu et al., 2020). Other chemical characteristics that have been reported to be related to bacterial community composition in composts include pH, organic matter and water soluble carbon, while fungal community composition can be related to organic carbon, water soluble carbon, and C/N (Zhang et al., 2011; Huhe et al., 2017; Sun et al., 2020; Wu et al., 2020). The chemical characteristics that were relatively strongly related to both bacterial and fungal community composition in this study were nitrate and Cl.

For nature management residues, feedstock-based subtypes were more difficult to distinguish based on microbiological community composition, with relative high dispersion between samples of feedstock-based subtypes. Miserez et al. (2019b) showed that management techniques, such as plaggen and chopping the heath vegetation, are an important determinant for chemical and physical characteristics of nature management residues. However, considerable variation in physical characteristics was seen between samples of chopped heath, which may be caused by variation in the amount of mineral material that is removed during chopping. Variation in management techniques may also cause variation in microbiological characteristics of feedstock-based subtypes observed in this study. Differences in bacterial community composition between management residues were mainly related to pH, while differences in fungal community composition were mainly related to hemicellulose content. Miserez et al. (2019b) reported that management residues show considerable differences in hemicellulose content. The fungal community may be influenced by hemicellulose, as saprotrophic fungi are efficient degraders of hemicellulose and other recalcitrant fractions of plant residues (van der Wal et al., 2013). No (bio)chemical characteristics were related to both bacterial and fungal community composition.

Previous studies already showed that composts and woody materials, such as wood fiber, display distinct microbial community profiles compared to peat (Green et al., 2004; Montagne et al., 2015, 2017). Montagne et al. (2015, 2017) reported that the microbial community in horticultural substrates is strongly dependent on substrate characteristics such as the origin of the material and physical structure due to the production process, resulting in globally distinct microbial communities in distinct types of substrates. Pot et al. (2022) showed that a microbial community diverging from that of peat-based substrates may be most favorable in disease suppressive growing media. Based on this information, for composts, green composts and woody composts have the most opportunity as these show the largest difference in either bacterial or fungal community compared to peat-based substrates. For management residues, woody fractions of composts showed the largest difference in bacterial community composition as compared to peat-based substrates. Grass clippings and chopped heath showed the largest difference in fungal community composition compared to pure and limed peat-based substrates, respectively.

Second, to look more into detail in the differences between subtypes of composts and management residues, different microbiological characteristics that have been reported in literature as indicators for plant growth and health promotion in horticultural substates or soils were evaluated, i.e., presence of genera associated with known beneficial microorganisms, absence of genera with known pathogens, microbial diversity, functional diversity and metabolic activity, microbial biomass, and fungal to bacterial ratio.

Some subtypes of composts and management residues showed a significantly higher abundance for genera known to be associated with beneficial microorganisms. Compared to pure peat-based substrates, only green composts, woody composts, and woody fractions of composts showed a significant increase in the relative abundance of at least one genus associated with beneficial microorganisms. Compared to limed peat-based substrates, more subtypes showed a significant increase in these genera and more of these genera were significantly increased in relative abundance. Green composts, VGF composts, woody composts, grass clippings, and woody fractions of composts showed an increase in at least three beneficial genera. The higher abundance of these genera in composts and management residues may be a benefit as compared to peat-based substrates. Several studies have shown high abundances of plant growth promoting microorganisms and biocontrol agents, such as *Bacillus* spp., *Pseudomonas* spp., *Serratia* spp., *Paenibacullus* spp., and *Trichoderma* spp., in composts, leading to better plant growth and higher disease suppressiveness (Dukare et al., 2011; Chen et al., 2012; Antoniou et al., 2017; Lutz et al., 2020; Neher et al., 2022). Mendes et al. (2011) showed that the relative abundance of genera associated with beneficial microorganisms (i.e., significant higher abundance) is an important indicator for disease suppressiveness. The important remark should be made, however, that metabarcoding does not allow to reliably identify microorganisms at species level and that their function is unknown. It is therefore not sure if beneficial species or strains are present and functional in the samples. Further analysis, such as isolation of these strains, would therefore be necessary to confirm the presence of beneficial strains in the samples. However, studies have shown that relative abundances at genus level can also give an indication of disease suppression (Mendes et al., 2011; Liu et al., 2016, 2019).

Besides beneficial microorganisms, also pathogens may be present in composts and management residues, including human pathogens, belonging to genera such as *Salmonella, Escherichia, Klebsiella*, *Shigella*, and *Enterobacter*, or plant pathogens that can infect the plant roots *via* the growing medium, belonging to genera such as *Verticillium*, *Rhizoctonia*, *Sclerotinia,* and *Plasmodiaphora*. The genera *Klebsiella*, *Enterobacter,* and *Escherichia*/*Shigella* were present in some subtypes of peat-based substrates, composts, and management residues, specifically in pure peat-based substrates, green composts, grass clippings, and woody fractions of composts. However, again, the remark should be made that metabarcoding cannot confirm the presence and function of pathogenic strains in the samples. Further analysis, such as isolation *via* plating, would be necessary to confirm this. Moreover, several of the species included in these genera even have been shown to be non-pathogenic or even to have positive effects on plants. Several strains in the *Escherichia/Shigella* genus have been reported to be non-pathogenic (Welch, 2006; Liu, 2019). *Enterobacter* sp. and *Klebsiella* sp. have been reported to have plant growth promoting or disease suppressive effects in different plants (Chelius and Triplett, 2000; Ngamau et al., 2012; Marcos et al., 2015; Anzuay et al., 2017; Neher et al., 2022). Further analysis at species level would be needed to study the presence of pathogenic species. In addition, although relative abundances of some of these genera were significantly increased is subtypes in composts and management residues, they were in general found in very small abundances. Other genera that may include human and/or plant pathogens, such as *Salmonella, Verticillium*, *Rhizoctonia*, *Sclerotinia* or *Plasmodiaphora*, were not found in any of the subtypes. Based on those results, it may be concluded that the different subtypes of composts and management residues are relatively safe for use in substrates.

Based on estimations of bacterial diversity in composts and peat by Neher et al. (2013) and De Tender et al. (2016a), respectively, it was hypothesized that composts are more diverse than peat. Green composts and woody composts showed a significant higher bacterial diversity than pure peat-based substrates, while chopped heath showed a significant higher fungal diversity than pure peat-based substrates. However, none of the subtypes of composts or management residues showed a significantly higher bacterial or fungal diversity than limed peat-based substrates. A higher microbial diversity may be considered to be positive for the use in substrates, as this may outcompete pathogens by niche saturation, leading to a higher disease suppressiveness (Chaparro et al., 2012; van Elsas et al., 2012; Bongiorno et al., 2019). In addition, studies have also shown a positive effect of microbial diversity on plant growth (Wagg et al., 2011; Weidner et al., 2015; Kolton et al., 2017).

Subtypes of composts and management residues were expected to have a higher functional diversity and metabolic activity than peat-based substrates (Pane et al., 2011; Lutz et al., 2020). However, subtypes of composts and management residues did not show a significant higher functional diversity or metabolic activity than subtypes of peat-based substrates.

Vandecasteele et al. (2021) showed that composts and management residues in general have a higher microbial biomass compared to peat-based substrates. The present study showed that this is not the case for all subtypes of composts and management residues. For composts, only green composts and VGF composts showed a significantly higher microbial biomass than pure and limed peat-based substrates. For management residues, grass clippings and chopped heath showed a significant higher microbial biomass than pure and limed peat-based substrates. In addition, Vandecasteele et al. (2021) showed that management residues showed in general a higher fungal to bacterial ratio than peat-based substrates. However, for the subtypes of management residues, only grass clippings and chopped heath showed a significant higher fungal to bacterial ratio. A high microbial biomass and fungal to bacterial ratio may be related to higher disease suppression (Bongiorno et al., 2019; De Corato, 2020; Neher et al., 2022). Microbial biomass has also been associated with increased yield (Shen et al., 2016).

To assess if the different beneficial microbiological characteristics of subtypes of composts and management residues result in enhanced plant growth and/or disease suppression, the data of this study should be linked to plant-pathogens experiments in which different subtypes are used as a peat replacer.

Third, it was assessed if different types of composts and management residues can increase inoculation efficiency of *T. harzianum*, a biocontrol fungus, and which microbiological characteristics of composts and management residues can be used to predict inoculation efficiency. Joos et al. (2020) showed that different composts are suitable carrier media for *T. harzianum*. In addition, Vandecasteele et al. (2021) showed that composts have a significant higher inoculation efficiency than peat-based substrates. However, when the subtypes of composts or management residues were compared to pure and limed peat-based substrates, no significant differences were found in inoculation efficiency. A possible explanation for this may be that there were no significant differences in the organic matter content between the subtypes of composts and management residues, which is positively correlated with the survival rate of *T. harizanum* (Kibaki et al., 2006). Net inoculation of *T. harzianum* was significantly correlated with initial microbiological characteristics of composts and management residues. For composts, the initial biomass of non-specific bacteria, Gram-positive bacteria, Actinomycetes, Gram-negative bacteria, fungi, and the total initial microbial biomass was significantly correlated with net inoculation of the biocontrol fungus. Fungal biomass showed the strongest correlation. For management residues, fungal biomass was significantly correlated with net inoculation of the biocontrol fungus. These results show that fungal biomass may be a suitable predictor for inoculation efficiency with *T. harzianum* for both composts and management residues. This may be due to lower competition for nutrients and niches in substrates with a low initial fungal biomass (Fliessbach et al., 2009; Quiza et al., 2015). In further research, the relation between fungal biomass and inoculation efficiency of a biocontrol fungus could be studied for different biocontrol products and in other horticultural substrates.

## Conclusion

For composts and peat-based substrates, a classification based on feedstock is relevant for bacterial and fungal community compositions, while for nature management residues feedstock-based subtypes may be less relevant for microbiology, as these subtypes were more difficult to distinguish based on microbial community composition. For composts, differences in bacterial community composition were related to chlorine and nitrate, while fungal community composition was related to nitrate. Bacterial and fungal community composition between management residues were mainly related to pH and hemicellulose content, respectively. Based on the microbiological characteristics, the subtypes showing the most potential to enhance plant growth and/or health are green composts, VFG composts, and woody composts in horticultural substrates for non-acidophilic plants and grass clippings, chopped heath, and woody fractions of compost for horticultural substrates for calcifuge plants. Further research should link these data to plant-pathogen experiments. Fungal biomass may be a suitable predictor for inoculation efficiency with *T. harzianum* for both composts and management residues. Further research should focus on evaluating this for different biocontrol products and other horticultural substrates.

## Data availability statement

The datasets presented in this study can be found in online repositories. The names of the repository/repositories and accession number(s) can be found at: https://www.ncbi.nlm.nih.gov/, PRJNA624053; https://www.ncbi.nlm.nih.gov/, PRJNA715731; https://www.ncbi.nlm.nih.gov/, PRJNA767265.

## Author contributions

JD, BV, ID, KV, CT, and JC were involved in the design and supervision of the study. SO conducted the metabarcoding, PLFA analysis, and inoculation experiment. SP conducted the Biolog EcoPlates experiment, conducted the statistical analysis of the data, and wrote the first draft and finalized the manuscript. SO and SP conducted the bio-informatics of the NGS data. All authors contributed to the article and approved the submitted version.

## Funding

This work was supported by Flanders Innovation & Entrepreneurship (HBC.2017.0815) (Bi-o-ptimal@work – Sustainable cultivation in container and open field by using innovative and local materials with enhanced microbial life, ready for use and implementation by ornamental growers). CT received a grant of the Research Foundation Flanders (FWO) with application number (12S9418N). KV received an FWO sabbatical bench fee (number VWH-E1313-SAB/22/016).

## Conflict of interest

The authors declare that the research was conducted in the absence of any commercial or financial relationships that could be construed as a potential conflict of interest.

## Publisher’s note

All claims expressed in this article are solely those of the authors and do not necessarily represent those of their affiliated organizations, or those of the publisher, the editors and the reviewers. Any product that may be evaluated in this article, or claim that may be made by its manufacturer, is not guaranteed or endorsed by the publisher.
