# Supplementary material for: Elucidating the microbiome of the sustainable peat replacers composts and nature management residues
Source: Front Microbiol. 2022 Sep 26;13:983855. doi: 10.3389/fmicb.2022.983855 (PMC9555241; doi:10.3389/fmicb.2022.983855)
Supplement: Supplementary file 1 [file Data_Sheet_1.PDF]

Supplementary files - Elucidating the microbiome of the sustainable peat replacers composts and nature management residues

Steffi Pot, Caroline De Tender, Sarah Ommeslag, Ilse Delcour, Johan Ceusters, Bart Vandecasteele, Jane Debode, Karen Vancampenhout

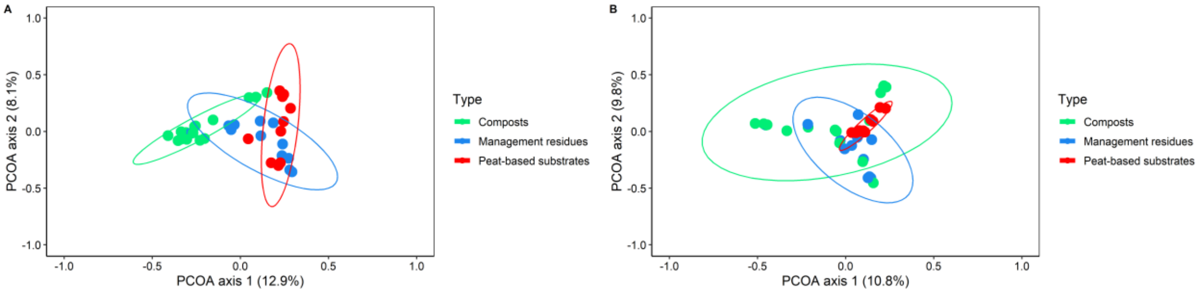

Supplementary Figure S1. Shifts in bacterial (A) and fungal (B) community composition between peat-based substrates, composts and management residues. Both figures representPrincipal Coordinate Analysis (PCoA) profiles of pairwise community dissimilarity (Bray-Curtis) indices, based on either 16S V3-V4 rRNA gene or ITS2 gene sequencing data for bacteria and fungi respectively. Colours indicate the three different types of biomass.

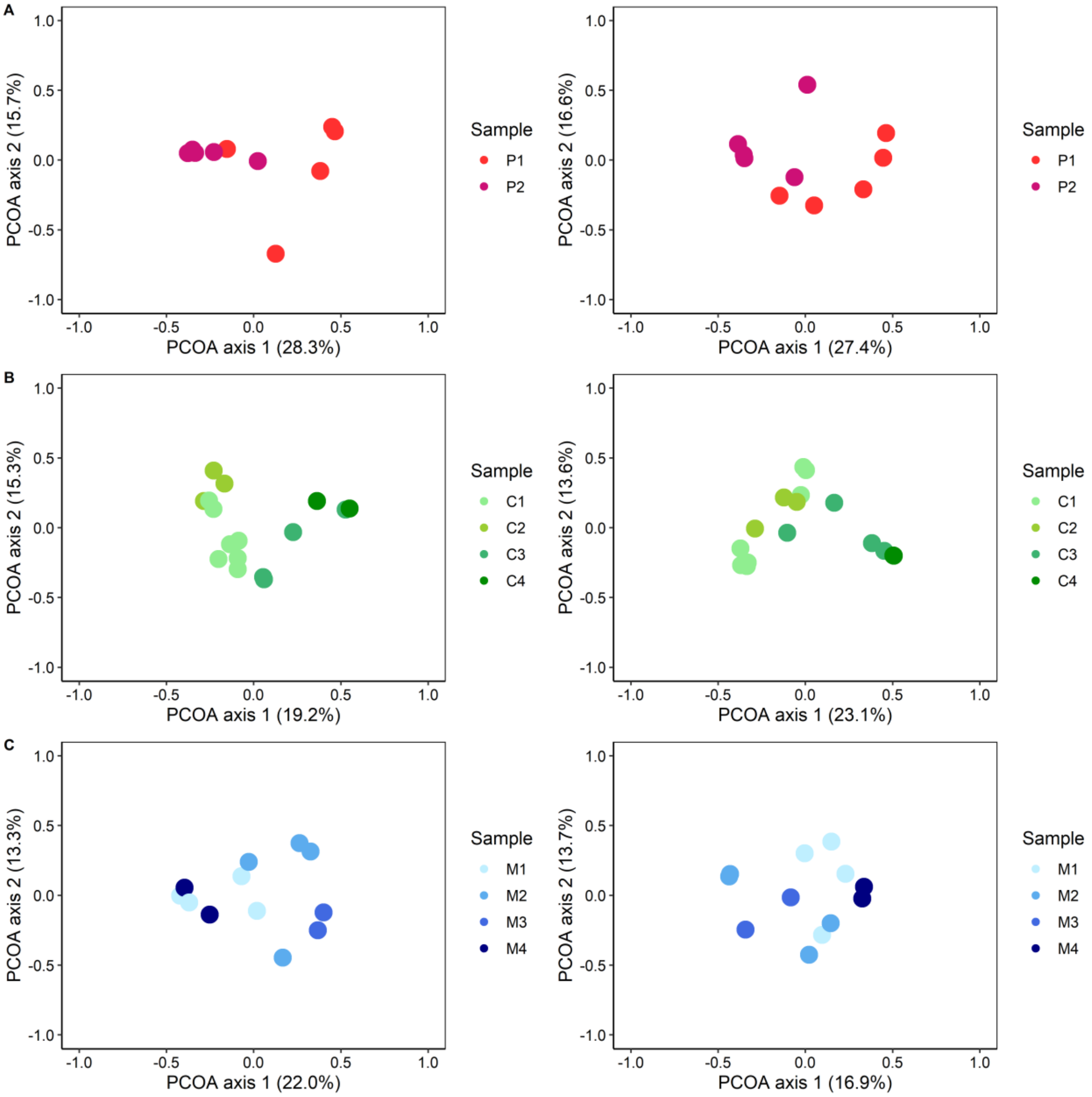

Supplementary Figure S2. Shifts in bacterial and fungal community composition between subtypes in peat-based substrates, composts and management residues. Principal Coordinate Analysis (PCoA) profile of pairwise community dissimilarity (Bray-Curtis) indices of on the right, bacterial (16S V3-V4 rRNA gene) ,and on the left, fungal (ITS2) sequencing data of (A) peat-based substrates, (B) composts, and (C) management residues. Colours indicate the subtypes of peat-based substrates (P1-P2), composts (C1-C4) or management residues (M1-M4). P1 = pure peat-based substrates (n = 5); P2 = limed peat-based substrates (n = 5); C1 = green composts (n = 7); C2 = VFG composts (n = 3); C3 = woody composts (n = 4); C4 = peat composts (n = 2); M1 = grass clippings (n = 4); M2 = chopped heath (n = 4); M3 = forest sods (n = 2); M4 = woody fractions of composts (n = 2).

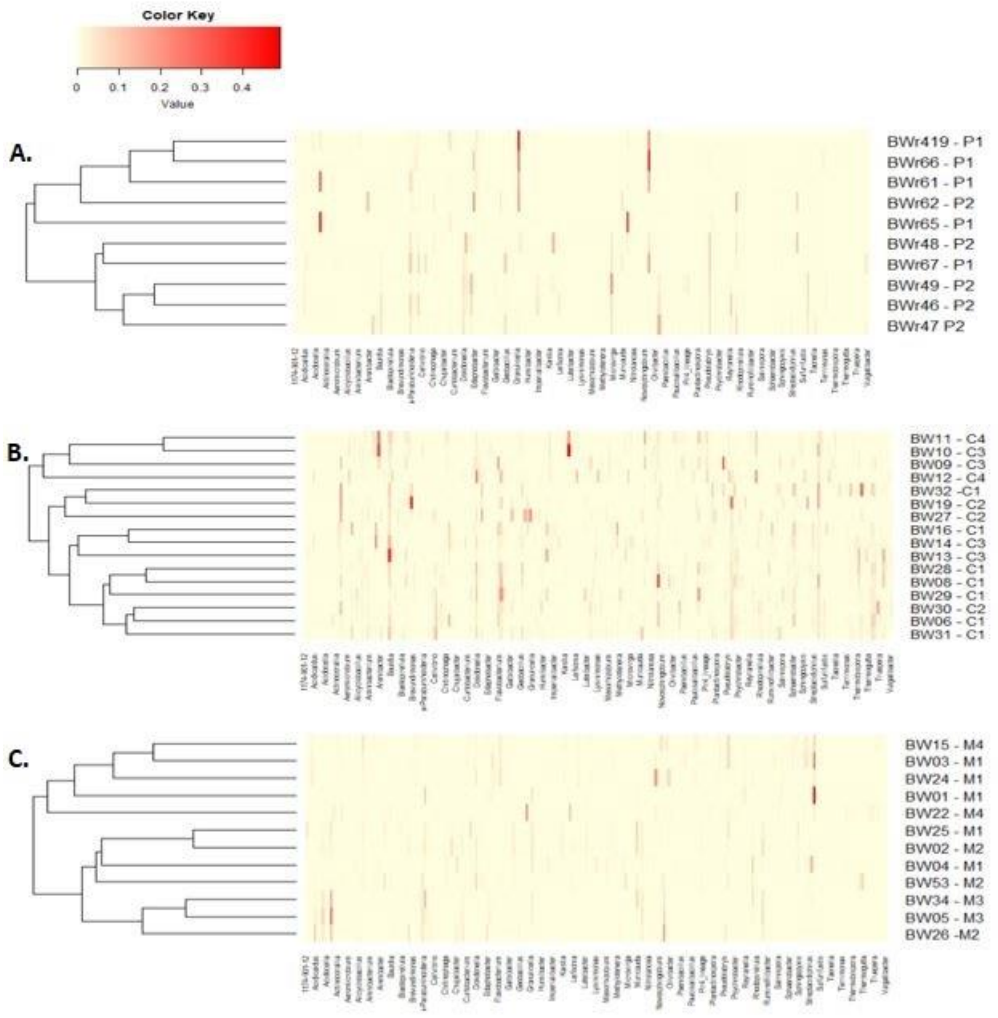

Supplementary Figure S3. Heatmap and clustering of the different samples of peat-based substrates (A), composts (B) and management residues (C) based on bacterial genera (16S V3-V4 rRNA gene) that showed a relative abundance of 1% in at least one sample. P1 = pure peat-based substrates (n = 5); P2 = limed peat-based substrates (n = 5); C1 = green composts (n = 7); C2 = VFG composts (n = 3); C3 = woody composts (n = 4); C4 = peat composts (n = 2); M1 = grass clippings (n = 4); M2 = chopped heath (n = 4); M3 = forest sods (n = 2); M4 = woody fractions of composts (n = 2).

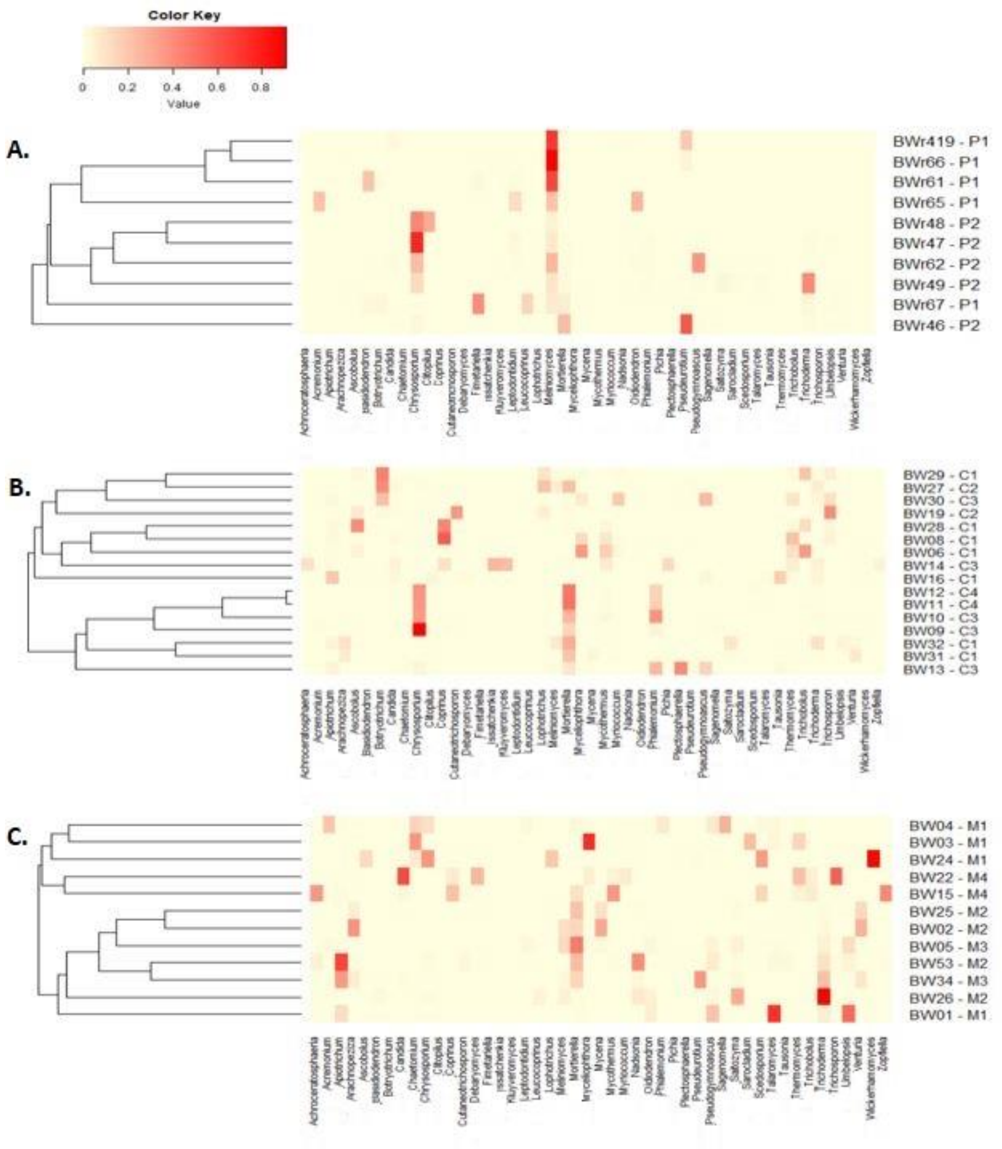

Supplementary Figure S4. Heatmap and clustering of the different samples of peat-based substrates (A), composts (B) and management residues (C) based on fungal genera (ITS2) that showed a relative abundance of 1% in at least one sample. P1 = pure peat-based substrates (n = 5); P2 = limed peat-based substrates (n = 5); C1 = green composts (n = 7); C2 = VFG composts (n = 3); C3 = woody composts (n = 4); C4 = peat composts (n = 2); M1 = grass clippings (n = 4); M2 = chopped heath (n = 4); M3 = forest sods (n = 2); M4 = woody fractions of composts (n = 2).

Supplementary Table S1. Associations between bacterial (left) and fungal (right) community composition and chemical characteristics and biomass of microbial groups in (A) peat-based substrates, (B) composts, (C) management residues. Bold indicates a significant association. (x and y: respective coordinates on the PCoA plot in the PCoA1 and PCoA2 axes; r^2: determination coefficient; EC: electrical conductivity, N_{min}: mineral N = NO_3–N + NH_4–N, OM: organic matter, DM: dry matter, P_{water}: water-extractable P, C_{water}: water-extractable C, N_{immob}: N immobilization, OUR: oxygen uptake rate)

A.

	x	y	r2	P-value
Cellulose (%/OM)	0.97	-0.26	0.33	0.75
Hemicellulose (%/OM)	0.93	-0.36	0.42	0.75
Lignin (%/OM)	-0.93	0.36	0.97	0.13
pH-H_2O (-)	0.98	-0.18	1.00	0.08
EC (µS/cm)	-0.97	0.23	0.39	0.75
NO_3-N (mg/l substrate)	-0.98	0.21	0.98	0.25
NH_4-N (mg/l substrate)	-0.97	0.26	0.94	0.25
N_{min} (mg/l substrate)	-0.98	0.21	0.94	0.25
SO_4 (mg/l substrate)	-0.97	0.26	0.99	0.17
Cl (mg/l substrate)	-0.94	0.33	0.90	0.42
OM (%/DM)	0.93	-0.38	0.94	0.21
P_{water} (mg/l substrate)	-0.93	0.38	0.74	0.50
C_{water} (mg/l substrate)	-0.74	0.67	0.95	0.13
C/N	0.93	-0.37	0.97	0.21
N_{immob} (%)	-0.96	0.28	0.24	0.83
OUR (mmol O2/kg OM/hr)	-0.95	0.31	0.49	0.67
Cumulative CO_2 release (mol CO2/ kg OM)	-0.88	0.47	0.88	0.42

	x	y	r2	P-value
Cellulose (%/OM)	0.81	-0.58	0.98	0.08
Hemicellulose (%/OM)	0.68	-0.73	0.92	0.25
Lignin (%/OM)	-0.48	0.88	0.08	1.00
pH-H_2O (-)	0.99	-0.11	0.97	0.13
EC (µS/cm)	-0.83	0.56	0.98	0.13
NO_3-N (mg/l substrate)	-0.95	0.30	0.98	0.25
NH_4-N (mg/l substrate)	-0.92	0.39	0.95	0.17
N_{min} (mg/l substrate)	-0.93	0.36	0.99	0.17
SO_4 (mg/l substrate)	-0.96	0.29	0.91	0.25
Cl (mg/l substrate)	-0.99	0.17	0.18	1.00
OM (%/DM)	-0.95	-0.31	0.30	0.92
P_{water} (mg/l substrate)	0.92	-0.39	0.22	1.00
C_{water} (mg/l substrate)	0.97	-0.22	0.94	0.25
C/N	0.48	-0.88	0.42	0.79
**N_{immob} (%)**	-0.80	0.61	0.99	0.04
OUR (mmol O2/kg OM/hr)	0.33	-0.94	0.48	0.75
Cumulative CO_2 release (mol CO2/ kg OM)	0.20	0.98	0.97	0.21

B.

	x	y	r2	P-value
Cellulose (%/OM)	0.81	-0.59	0.19	0.25
Hemicellulose (%/OM)	1.00	-0.08	0.35	0.07
Lignin (%/OM)	0.98	-0.19	0.17	0.31
**pH-H_2O (-)**	-0.96	0.29	0.67	0.00
**EC (µS/cm)**	0.00	1.00	0.60	0.00
**NO_3-N (mg/l substrate)**	0.69	0.73	0.68	0.00
**NH_4-N (mg/l substrate)**	-0.42	0.91	0.32	0.05
**N_{min} (mg/l substrate)**	0.36	0.93	0.56	0.00
**SO_4 (mg/l substrate)**	0.67	0.74	0.50	0.02
**Cl (mg/l substrate)**	-0.72	0.70	0.85	0.00
OM (%/DM)	0.97	-0.24	0.32	0.10
**P_{water} (mg/l substrate)**	0.89	0.46	0.41	0.03
C_{water} (mg/l substrate)	-0.98	-0.17	0.25	0.17
**C/N**	0.57	-0.82	0.37	0.03
N_{immob} (%)	0.35	-0.94	0.26	0.15
**OUR (mmol O2/kg OM/hr)**	-0.60	-0.80	0.37	0.05
**Cumulative CO_2 release (mol CO2/ kg OM)**	-0.44	-0.90	0.41	0.04

	x	y	r2	P-value
Cellulose (%/OM)	0.85	-0.52	0.17	0.28
**Hemicellulose (%/OM)**	0.98	-0.21	0.46	0.02
Lignin (%/OM)	0.95	-0.30	0.21	0.20
**pH-H_2O (-)**	-0.90	0.43	0.53	0.01
EC (µS/cm)	0.61	0.79	0.17	0.28
**NO_3-N (mg/l substrate)**	0.99	-0.16	0.57	0.01
NH_4-N (mg/l substrate)	-0.12	0.99	0.15	0.35
N_{min} (mg/l substrate)	0.94	0.33	0.29	0.11
**SO_4 (mg/l substrate)**	0.89	-0.46	0.40	0.05
**Cl (mg/l substrate)**	-0.45	0.89	0.52	0.01
OM (%/DM)	0.92	-0.39	0.35	0.05
P_{water} (mg/l substrate)	1.00	-0.04	0.32	0.07
C_{water} (mg/l substrate)	-0.83	0.55	0.25	0.15
C/N	0.93	-0.36	0.13	0.43
N_{immob} (%)	1.00	-0.08	0.03	0.81
**OUR (mmol O2/kg OM/hr)**	-0.90	-0.43	0.37	0.05
Cumulative CO_2 release (mol CO2/ kg OM)	-1.00	-0.05	0.18	0.27

C.

	x	y	r2	P-value
Cellulose (%/OM)	-0.93	0.36	0.43	0.10
Hemicellulose (%/OM)	-0.96	0.29	0.46	0.06
Lignin (%/OM)	0.85	0.53	0.08	0.69
**pH-H_2O (-)**	-0.92	0.40	0.74	0.01
EC (µS/cm)	-0.74	-0.67	0.42	0.06
NO_3-N (mg/l substrate)	0.22	-0.98	0.29	0.21
NH_4-N (mg/l substrate)	-0.09	-1.00	0.40	0.06
N_{min} (mg/l substrate)	0.05	-1.00	0.38	0.10
SO_4 (mg/l substrate)	-0.78	-0.62	0.42	0.09
Cl (mg/l substrate)	-0.90	-0.43	0.27	0.23
OM (%/DM)	-0.95	0.30	0.26	0.27
P_{water} (mg/l substrate)	-0.99	0.15	0.20	0.36
**C_{water} (mg/l substrate)**	-0.98	-0.18	0.48	0.04
C/N	-0.75	0.66	0.19	0.42
N_{immob} (%)	-0.14	0.99	0.30	0.22
OUR (mmol O2/kg OM/hr)	-0.82	0.58	0.45	0.06
Cumulative CO_2 release (mol CO2/ kg OM)	-0.91	0.41	0.51	0.07

	x	y	r2	P-value
Cellulose (%/OM)	0.65	0.76	0.32	0.16
**Hemicellulose (%/OM)**	0.36	0.93	0.51	0.02
Lignin (%/OM)	-0.96	-0.28	0.02	0.91
pH-H_2O (-)	0.80	0.60	0.40	0.09
EC (µS/cm)	0.99	0.15	0.45	0.06
NO_3-N (mg/l substrate)	0.77	-0.63	0.03	0.87
NH_4-N (mg/l substrate)	0.95	-0.30	0.13	0.67
N_{min} (mg/l substrate)	0.91	-0.42	0.08	0.77
SO_4 (mg/l substrate)	0.99	0.12	0.43	0.08
Cl (mg/l substrate)	0.97	0.24	0.31	0.16
OM (%/DM)	0.47	0.88	0.33	0.14
P_{water} (mg/l substrate)	0.88	-0.47	0.27	0.27
C_{water} (mg/l substrate)	0.90	0.43	0.44	0.05
C/N	1.00	0.10	0.07	0.81
N_{immob} (%)	-0.50	-0.86	0.01	0.95
OUR (mmol O2/kg OM/hr)	0.24	0.97	0.35	0.12
Cumulative CO_2 release (mol CO2/ kg OM)	0.57	0.82	0.40	0.11

Supplementary Table S2 Shifts in bacterial community distribution between subtypes of peat-based substrates (P1-P2) and subtypes of composts (C1-C4) and subtypes of management residues (M1-M4). (A) Total number of significantly altered bacterial taxa, number of significantly more abundant bacterial taxa and number of significantly less abundant bacterial taxa between subtype P1 of peat-based substrates on one hand and subtypes of composts and management residues on the other hand at phylum, family and genus level. (B) Total number of significantly altered bacterial taxa, number of significantly more abundant bacterial taxa and number of significantly less abundant bacterial taxa between subtype P2 of peat-based substrates on one hand and subtypes of composts and management residues on the other hand at phylum, family and genus level. P1 = pure peat-based substrates (n = 5); P2 = limed peat-based substrates (n = 5); C1 = green composts (n = 7); C2 = VFG composts (n = 3); C3 = woody composts (n = 4); C4 = peat composts (n = 2); M1 = grass clippings (n = 4); M2 = chopped heath (n = 4); M3 = forest sods (n = 2); M4 = woody fractions of composts (n = 2).

A.

	**Composts**				**Management residues**			
	C1	C2	C3	C4	M1	M2	M3	M4
Reference	P1	P1	P1	P1	P1	P1	P1	P1
**PHYLA (n = 26)**								
Total significantly altered	11	8	9	2	1	1	1	1
Significantly more abundant	8	6	6	1	0	1	0	0
Significantly less abundant	3	2	3	1	1	0	1	1
**FAMILIES (n = 218)**								
Total significantly altered	77	49	42	13	16	2	0	16
Significantly more abundant	55	30	30	12	9	2	0	15
Significantly less abundant	22	19	12	1	7	0	0	1
**GENERA (n = 476)**								
Total significantly altered	76	18	56	0	13	0	0	26
Significantly more abundant	41	7	41	0	8	0	0	25
Significantly less abundant	35	11	15	0	5	0	0	1

B.

	**Composts**				**Management residues**			
	C1	C2	C3	C4	M1	M2	M3	M4
Reference	P2	P2	P2	P2	P2	P2	P2	P2
**PHYLA (n = 7)**								
Total significantly altered	13	12	7	2	4	4	3	2
Significantly more abundant	10	9	6	2	2	3	2	2
Significantly less abundant	3	3	1	0	2	1	1	0
**FAMILIES (n = 142)**								
Total significantly altered	148	111	75	25	41	19	0	55
Significantly more abundant	111	82	67	25	26	17	0	53
Significantly less abundant	37	29	8	0	15	2	0	2
**GENERA (n = 238)**								
Total significantly altered	268	145	157	1	56	15	0	129
Significantly more abundant	208	121	148	1	51	13	0	124
Significantly less abundant	60	24	9	0	5	2	0	5

Supplementary Table S3. Shifts in fungal community distribution between subtypes of peat-based substrates (P1-P2) and subtypes of composts (C1-C4) and subtypes of management residues (M1-M4). (A) Total number of significantly altered fungal taxa, number of significantly more abundant fungal taxa and number of significantly less abundant fungal taxa between subtype P1 of peat-based substrates on one hand and subtypes of composts and management residues on the other hand at phylum, family and genus level. (B) Total number of significantly altered fungal taxa, number of significantly more abundant fungal taxa and number of significantly less abundant fungal taxa between subtype P2 of peat-based substrates on one hand and subtypes of composts and management residues on the other hand at phylum, family and genus level. P1 = pure peat-based substrates (n = 5); P2 = limed peat-based substrates (n = 5); C1 = green composts (n = 7); C2 = VFG composts (n = 3); C3 = woody composts (n = 4); C4 = peat composts (n = 2); M1 = grass clippings (n = 4); M2 = chopped heath (n = 4); M3 = forest sods (n = 2); M4 = woody fractions of composts (n = 2).

A.

	**Composts**				**Management residues**			
	C1	C2	C3	C4	M1	M2	M3	M4
Reference	P1	P1	P1	P1	P1	P1	P1	P1
**PHYLA**								
Total significantly altered	0	2	3	2	2	0	1	1
Significantly more abundant	0	2	2	2	1	0	1	1
Significantly less abundant	0	0	1	0	1	0	0	0
**FAMILIES**								
Total significantly altered	16	0	14	0	6	3	0	4
Significantly more abundant	7	0	10	0	3	2	0	3
Significantly less abundant	9	0	4	0	3	1	0	1
**GENERA**								
Total significantly altered	3	0	7	0	8	0	0	2
Significantly more abundant	1	0	6	0	4	0	0	2
Significantly less abundant	2	0	1	0	4	0	0	0

B.

	**Composts**				**Management residues**			
	C1	C2	C3	C4	M1	M2	M3	M4
Reference	P2	P2	P2	P2	P2	P2	P2	P2
PHYLA								
Total significantly altered	0	0	2	1	3	0	0	0
Significantly more abundant	0	0	1	1	1	0	0	0
Significantly less abundant	0	0	1	0	2	0	0	0
FAMILIES								
Total significantly altered	19	0	8	0	16	9	1	3
Significantly more abundant	13	0	7	0	11	7	1	3
Significantly less abundant	6	0	1	0	5	2	0	0
GENERA								
Total significantly altered	0	0	6	0	2	14	0	1
Significantly more abundant	0	0	6	0	1	13	0	1
Significantly less abundant	0	0	0	0	1	1	0	0

Supplementary Table S4 Relative abundance of differential abundant bacterial (16S rRNA V3-V4) genera in the subtypes of composts (C1-C4) as compared to the two subtypes of peat-based substrates (P1 and P2) that represent at least 1% in one of the subtypes. Asterisks indicate a significant difference in the relative abundance as compared to subtype P1 of peat-based substrates. Hasthag indicates a significant difference in the relative abundance as compared to subtype P2 of peat-based substrates. P1 = pure peat-based substrates (n = 5); P2 = limed peat-based substrates (n = 5); C1 = green composts (n = 7); C2 = VFG composts (n = 3); C3 = woody composts (n = 4); C4 = peat composts (n = 2).

Genus	P1	P2	C1	C2	C3	C4
Acidibacter	2.32E-03 ± 1.12E-03	6.54E-03 ± 3.58E-03	4.43E-04 ± 2.59E-04	0.00E00 ± 0.00E00 #	4.71E-03 ± 1.79E-03	7.72E-03 ± 6.60E-03
Acidipila	3.63E-03 ± 1.73E-03	2.82E-04 ± 1.32E-04	0.00E00 ± 0.00E00 * #	0.00E00 ± 0.00E00 *	0.00E00 ± 0.00E00 *	0.00E00 ± 0.00E00
Acidothermus	1.28E-01 ± 8.28E-02	1.75E-03 ± 6.12E-04	0.00E00 ± 0.00E00 * #	0.00E00 ± 0.00E00 * #	2.87E-04 ± 2.87E-04 *	0.00E00 ± 0.00E00
Acinetobacter	0.00E00 ± 0.00E00	0.00E00 ± 0.00E00	1.71E-03 ± 1.23E-03 * #	2.10E-05 ± 2.10E-05	1.19E-04 ± 7.21E-05 #	7.16E-04 ± 7.16E-04
Actinomadura	5.31E-04 ± 3.74E-04	0.00E00 ± 0.00E00	1.99E-02 ± 1.04E-02 #	5.89E-02 ± 1.27E-02 #	1.15E-02 ± 5.64E-03 #	1.20E-03 ± 1.20E-03
Aeromicrobium	0.00E00 ± 0.00E00	4.70E-04 ± 3.34E-04	1.67E-03 ± 4.60E-04 *	1.60E-03 ± 7.16E-04	2.63E-03 ± 2.30E-03	1.19E-02 ± 7.08E-03
Aestuariicella	1.83E-04 ± 1.23E-04	0.00E00 ± 0.00E00	1.14E-02 ± 5.43E-03 #	5.14E-03 ± 2.27E-03 #	0.00E00 ± 0.00E00	0.00E00 ± 0.00E00
Allorhizobium	1.72E-04 ± 1.43E-04	4.86E-05 ± 3.42E-05	4.53E-03 ± 1.18E-03 #	5.38E-03 ± 2.50E-03 #	4.71E-03 ± 8.27E-04 #	1.95E-03 ± 8.91E-04
Anaerolinea	5.88E-05 ± 5.88E-05	0.00E00 ± 0.00E00	4.94E-03 ± 4.53E-03 #	3.60E-05 ± 3.60E-05	1.55E-02 ± 1.51E-02 * #	5.25E-05 ± 5.25E-05
Arachidicoccus	3.04E-04 ± 3.04E-04	1.76E-02 ± 1.76E-02	9.40E-06 ± 9.40E-06 #	0.00E00 ± 0.00E00 #	3.73E-02 ± 3.16E-02	4.61E-02 ± 4.61E-02
Asticcacaulis	8.62E-06 ± 8.62E-06	9.76E-03 ± 8.55E-03	1.42E-04 ± 6.91E-05 #	0.00E00 ± 0.00E00 #	2.62E-04 ± 1.83E-04	4.04E-03 ± 8.63E-04
Bacillus	1.94E-04 ± 1.94E-04	2.52E-04 ± 2.37E-04	2.30E-02 ± 5.13E-03 #	1.76E-02 ± 5.22E-03 #	5.36E-02 ± 3.22E-02 * #	1.66E-02 ± 1.45E-02
Bauldia	1.68E-03 ± 1.62E-03	1.80E-02 ± 7.74E-03	1.66E-04 ± 8.62E-05 #	2.06E-04 ± 1.16E-04	2.35E-03 ± 1.85E-03	2.14E-02 ± 4.44E-03
Bradyrhizobium	4.67E-04 ± 4.67E-04	1.24E-03 ± 7.66E-04	0.00E00 ± 0.00E00 * #	0.00E00 ± 0.00E00 #	2.80E-03 ± 2.80E-03	7.00E-03 ± 6.17E-03
Brevibacterium	6.75E-05 ± 6.75E-05	0.00E00 ± 0.00E00	3.89E-03 ± 3.75E-03 #	5.35E-02 ± 5.06E-02 #	1.63E-04 ± 1.06E-04 #	1.03E-03 ± 7.86E-04
Bryobacter	1.66E-02 ± 8.82E-03	1.85E-02 ± 5.95E-03	3.81E-05 ± 2.89E-05 * #	0.00E00 ± 0.00E00 * #	3.52E-03 ± 1.89E-03	1.32E-03 ± 9.27E-04
Burkholderia	4.95E-03 ± 2.64E-03	3.96E-03 ± 2.98E-03	0.00E00 ± 0.00E00 * #	0.00E00 ± 0.00E00 * #	5.14E-04 ± 3.25E-04	0.00E00 ± 0.00E00
Candidatus_Solibacter	1.06E-02 ± 5.83E-03	1.44E-02 ± 7.51E-03	0.00E00 ± 0.00E00 * #	0.00E00 ± 0.00E00 * #	1.55E-03 ± 1.27E-03	1.28E-03 ± 8.49E-04
Cellvibrio	2.30E-04 ± 1.66E-04	0.00E00 ± 0.00E00	1.70E-02 ± 7.26E-03 #	1.11E-02 ± 6.89E-03 #	3.20E-04 ± 2.88E-04 #	1.53E-03 ± 1.53E-03
Chthoniobacter	1.01E-04 ± 1.01E-04	0.00E00 ± 0.00E00	1.18E-03 ± 5.77E-04 #	1.80E-05 ± 1.80E-05	1.78E-03 ± 1.23E-03 #	2.73E-04 ± 2.73E-04
Conexibacter	1.27E-02 ± 5.98E-03	1.82E-03 ± 9.82E-04	0.00E00 ± 0.00E00 * #	5.41E-05 ± 2.71E-05	1.79E-04 ± 8.71E-05	2.53E-03 ± 2.53E-03
Cytophaga	3.07E-04 ± 1.96E-04	0.00E00 ± 0.00E00	3.44E-03 ± 1.70E-03 #	6.47E-04 ± 2.31E-04 #	5.58E-05 ± 4.64E-05	0.00E00 ± 0.00E00
Dokdonella	1.61E-03 ± 1.61E-03	1.89E-02 ± 9.72E-03	7.16E-05 ± 3.79E-05 #	1.35E-04 ± 1.35E-04	2.66E-03 ± 5.21E-04	7.17E-03 ± 5.78E-03
Domibacillus	2.25E-04 ± 2.25E-04	0.00E00 ± 0.00E00	7.51E-03 ± 5.08E-03 #	2.46E-02 ± 1.58E-02 #	1.81E-05 ± 1.81E-05	0.00E00 ± 0.00E00
Dongia	0.00E00 ± 0.00E00	2.36E-02 ± 1.27E-02	0.00E00 ± 0.00E00 #	0.00E00 ± 0.00E00 #	1.86E-03 ± 4.24E-04 *	3.26E-03 ± 1.47E-04
Dyella	1.72E-02 ± 1.39E-02	3.59E-02 ± 3.28E-02	0.00E00 ± 0.00E00 * #	0.00E00 ± 0.00E00 * #	9.57E-04 ± 8.50E-04	2.73E-04 ± 1.04E-04
Edaphobacter	8.40E-03 ± 4.00E-03	3.77E-04 ± 2.34E-04	0.00E00 ± 0.00E00 * #	0.00E00 ± 0.00E00 *	2.16E-03 ± 2.16E-03	7.93E-04 ± 7.93E-04
Enterobacter	0.00E00 ± 0.00E00	0.00E00 ± 0.00E00	1.12E-04 ± 1.12E-04 #	0.00E00 ± 0.00E00	0.00E00 ± 0.00E00	0.00E00 ± 0.00E00
Filomicrobium	3.70E-04 ± 2.35E-04	0.00E00 ± 0.00E00	1.51E-02 ± 3.59E-03 #	5.28E-03 ± 4.70E-03 #	1.95E-02 ± 1.37E-02 #	0.00E00 ± 0.00E00
Flavobacterium	1.15E-03 ± 6.83E-04	0.00E00 ± 0.00E00	2.79E-02 ± 9.96E-03 #	1.64E-02 ± 7.83E-03 #	2.02E-03 ± 1.32E-03 #	1.87E-02 ± 1.86E-02 #
Galbibacter	2.81E-04 ± 2.81E-04	0.00E00 ± 0.00E00	1.48E-03 ± 1.35E-03 #	1.94E-02 ± 1.00E-02 #	3.73E-04 ± 3.67E-04 #	1.03E-03 ± 1.03E-03
Geobacillus	1.53E-04 ± 1.53E-04	0.00E00 ± 0.00E00	1.90E-02 ± 4.90E-03 #	2.86E-03 ± 1.81E-03 #	4.48E-03 ± 1.79E-03 #	1.30E-03 ± 1.30E-03
Gilvimarinus	1.19E-04 ± 1.19E-04	0.00E00 ± 0.00E00	5.31E-05 ± 5.31E-05	1.86E-02 ± 1.73E-02 #	0.00E00 ± 0.00E00	0.00E00 ± 0.00E00
Glutamicibacter	1.80E-04 ± 1.80E-04	0.00E00 ± 0.00E00	4.24E-03 ± 1.71E-03 #	1.31E-02 ± 5.87E-03 #	1.83E-04 ± 1.83E-04 #	2.86E-03 ± 2.86E-03
Glycomyces	5.21E-04 ± 3.45E-04	0.00E00 ± 0.00E00	3.24E-04 ± 2.46E-04 #	2.49E-02 ± 2.33E-02 #	0.00E00 ± 0.00E00 *	0.00E00 ± 0.00E00
Granulicella	1.10E-01 ± 4.97E-02	4.07E-02 ± 3.40E-02	0.00E00 ± 0.00E00 * #	0.00E00 ± 0.00E00 * #	0.00E00 ± 0.00E00 * #	1.23E-04 ± 1.23E-04
Hydrogenispora	0.00E00 ± 0.00E00	0.00E00 ± 0.00E00	5.76E-03 ± 3.28E-03 * #	4.21E-03 ± 1.47E-03 * #	1.46E-02 ± 1.36E-02 * #	2.52E-04 ± 2.52E-04
Kribbella	8.62E-06 ± 8.62E-06	1.60E-02 ± 1.22E-02	0.00E00 ± 0.00E00 #	0.00E00 ± 0.00E00 #	4.46E-02 ± 4.46E-02 *	3.09E-02 ± 3.09E-02
Kurthia	0.00E00 ± 0.00E00	0.00E00 ± 0.00E00	3.22E-04 ± 1.76E-04 #	0.00E00 ± 0.00E00	1.78E-04 ± 1.78E-04 #	0.00E00 ± 0.00E00
Lelliottia	0.00E00 ± 0.00E00	0.00E00 ± 0.00E00	1.90E-04 ± 1.66E-04 #	0.00E00 ± 0.00E00	0.00E00 ± 0.00E00	6.78E-04 ± 6.78E-04
Lysinimonas	0.00E00 ± 0.00E00	8.99E-04 ± 5.76E-04	0.00E00 ± 0.00E00 #	0.00E00 ± 0.00E00	2.96E-04 ± 2.96E-04	1.64E-02 ± 1.29E-02
Micropepsis	4.26E-03 ± 4.26E-03	4.62E-02 ± 2.31E-02	0.00E00 ± 0.00E00 * #	0.00E00 ± 0.00E00 #	8.34E-04 ± 5.37E-04	1.19E-02 ± 1.09E-02
Mucilaginibacter	3.04E-03 ± 1.84E-03	1.06E-02 ± 9.07E-03	1.57E-05 ± 1.57E-05 * #	0.00E00 ± 0.00E00 #	0.00E00 ± 0.00E00 * #	0.00E00 ± 0.00E00
Mycobacterium	7.69E-02 ± 7.17E-02	5.14E-03 ± 2.82E-03	4.15E-03 ± 1.08E-03 *	2.86E-03 ± 2.72E-04	1.26E-02 ± 5.69E-03	3.06E-02 ± 2.92E-02
Nonomuraea	0.00E00 ± 0.00E00	0.00E00 ± 0.00E00	2.84E-02 ± 1.84E-02 * #	1.34E-02 ± 6.59E-03 * #	6.08E-03 ± 1.13E-03 * #	7.65E-03 ± 7.65E-03
Occallatibacter	1.24E-01 ± 4.84E-02	7.72E-03 ± 4.35E-03	0.00E00 ± 0.00E00 * #	0.00E00 ± 0.00E00 * #	0.00E00 ± 0.00E00 * #	1.79E-04 ± 1.79E-04
Ochrobactrum	0.00E00 ± 0.00E00	0.00E00 ± 0.00E00	4.68E-03 ± 3.56E-03 * #	7.41E-04 ± 4.89E-04 #	1.97E-04 ± 1.97E-04 #	0.00E00 ± 0.00E00
Olivibacter	0.00E00 ± 0.00E00	0.00E00 ± 0.00E00	2.41E-03 ± 2.17E-03 * #	0.00E00 ± 0.00E00	1.19E-03 ± 5.98E-04 #	8.56E-03 ± 8.56E-03
Opitutus	9.81E-05 ± 9.81E-05	3.02E-02 ± 1.88E-02	2.83E-04 ± 1.90E-04 #	0.00E00 ± 0.00E00 #	1.54E-03 ± 7.67E-04	7.06E-03 ± 1.14E-03
Ornithinicoccus	0.00E00 ± 0.00E00	0.00E00 ± 0.00E00	4.20E-03 ± 1.72E-03 * #	1.09E-02 ± 1.04E-02 * #	2.42E-05 ± 2.42E-05	0.00E00 ± 0.00E00
Pedobacter	1.16E-03 ± 7.57E-04	8.15E-05 ± 8.15E-05	1.50E-02 ± 8.79E-03 #	7.55E-03 ± 3.50E-03 #	4.97E-03 ± 3.38E-03 #	1.71E-02 ± 1.64E-02
Planifilum	4.28E-05 ± 4.28E-05	0.00E00 ± 0.00E00	1.11E-02 ± 5.20E-03 #	9.67E-03 ± 3.62E-03 #	0.00E00 ± 0.00E00	0.00E00 ± 0.00E00
Promicromonospora	1.56E-04 ± 9.99E-05	2.51E-05 ± 2.51E-05	5.32E-03 ± 4.31E-03 #	9.91E-04 ± 6.31E-04	2.39E-02 ± 2.38E-02 #	1.76E-03 ± 8.51E-04
Pseudolabrys	9.46E-03 ± 7.20E-03	3.15E-02 ± 7.90E-03	1.10E-04 ± 1.10E-04 * #	0.00E00 ± 0.00E00 * #	3.26E-03 ± 1.88E-03	1.71E-02 ± 9.13E-03
Pseudomonas	3.72E-03 ± 2.62E-03	2.61E-04 ± 1.69E-04	1.58E-02 ± 2.87E-03 #	4.53E-02 ± 2.99E-02 #	4.92E-03 ± 2.52E-03 #	8.13E-03 ± 7.00E-03
Pseudonocardia	6.98E-05 ± 6.98E-05	1.86E-05 ± 1.86E-05	1.41E-02 ± 5.39E-03 #	5.25E-04 ± 5.25E-04	3.92E-03 ± 1.37E-03 #	9.25E-04 ± 9.25E-04
Pusillimonas	0.00E00 ± 0.00E00	0.00E00 ± 0.00E00	1.17E-03 ± 6.55E-04 #	1.00E-02 ± 7.14E-03 * #	0.00E00 ± 0.00E00	0.00E00 ± 0.00E00
Rahnella	0.00E00 ± 0.00E00	0.00E00 ± 0.00E00	1.04E-04 ± 5.71E-05 #	0.00E00 ± 0.00E00	0.00E00 ± 0.00E00	0.00E00 ± 0.00E00
Reyranella	8.55E-05 ± 8.55E-05	1.65E-02 ± 1.04E-02	1.11E-04 ± 6.30E-05 #	0.00E00 ± 0.00E00 #	1.40E-03 ± 7.96E-04	4.61E-03 ± 4.61E-03
Rhodanobacter	6.27E-03 ± 2.64E-03	3.97E-02 ± 2.28E-02	2.20E-04 ± 1.30E-04 * #	2.19E-04 ± 8.19E-05	8.02E-03 ± 7.36E-03	5.10E-02 ± 1.87E-02
Roseiarcus	9.28E-04 ± 2.62E-04	8.55E-04 ± 4.52E-04	2.53E-05 ± 2.53E-05 *	0.00E00 ± 0.00E00	0.00E00 ± 0.00E00 * #	0.00E00 ± 0.00E00
Sphaerobacter	2.41E-04 ± 2.41E-04	0.00E00 ± 0.00E00	1.75E-02 ± 4.99E-03 #	5.89E-03 ± 2.31E-03 #	7.33E-03 ± 2.24E-03 #	0.00E00 ± 0.00E00
Sphingobacterium	1.45E-04 ± 9.21E-05	0.00E00 ± 0.00E00	3.59E-03 ± 2.26E-03 #	3.06E-03 ± 2.79E-03 #	5.68E-05 ± 4.66E-05	0.00E00 ± 0.00E00
Sphingomonas	1.01E-03 ± 1.01E-03	1.86E-03 ± 1.45E-03	4.68E-04 ± 2.30E-04	0.00E00 ± 0.00E00 #	2.61E-03 ± 2.39E-03	3.52E-03 ± 2.91E-03
Sphingopyxis	0.00E00 ± 0.00E00	0.00E00 ± 0.00E00	1.28E-03 ± 3.89E-04 #	4.08E-04 ± 2.06E-04 #	5.50E-04 ± 2.12E-04 #	1.04E-02 ± 6.97E-03
Staphylococcus	0.00E00 ± 0.00E00	0.00E00 ± 0.00E00	5.76E-04 ± 4.28E-04 #	2.15E-02 ± 2.13E-02 * #	0.00E00 ± 0.00E00	5.43E-04 ± 4.37E-04

Stenotrophomonas	3.10E-05 ± 3.10E-05	0.00E00 ± 0.00E00	1.72E-03 ± 1.10E-03 #	0.00E00 ± 0.00E00	0.00E00 ± 0.00E00	1.04E-04 ± 1.04E-04
Streptacidiphilus	0.00E00 ± 0.00E00	7.06E-04 ± 4.33E-04	0.00E00 ± 0.00E00 #	0.00E00 ± 0.00E00	1.15E-03 ± 1.15E-03	8.41E-05 ± 8.41E-05
SWB02	4.62E-04 ± 4.62E-04	1.99E-02 ± 8.44E-03	0.00E00 ± 0.00E00 * #	0.00E00 ± 0.00E00 #	2.27E-03 ± 1.40E-03	1.31E-02 ± 1.31E-02
Thermobispora	0.00E00 ± 0.00E00	0.00E00 ± 0.00E00	1.13E-02 ± 5.91E-03 * #	4.40E-04 ± 3.35E-04 #	1.64E-02 ± 1.04E-02 * #	3.73E-04 ± 3.73E-04
Thermocrispum	4.05E-05 ± 4.05E-05	0.00E00 ± 0.00E00	2.04E-02 ± 1.54E-02 * #	4.54E-03 ± 3.83E-03 #	3.13E-04 ± 2.50E-04 #	0.00E00 ± 0.00E00
Thermogemmatispora	0.00E00 ± 0.00E00	0.00E00 ± 0.00E00	0.00E00 ± 0.00E00	0.00E00 ± 0.00E00	4.92E-03 ± 3.98E-03 * #	0.00E00 ± 0.00E00
Thermomonospora	1.80E-05 ± 1.80E-05	0.00E00 ± 0.00E00	1.85E-02 ± 3.57E-03 * #	4.25E-03 ± 3.00E-03 #	2.98E-03 ± 1.20E-03 #	0.00E00 ± 0.00E00
Thermopolyspora	0.00E00 ± 0.00E00	0.00E00 ± 0.00E00	1.32E-02 ± 2.63E-03 * #	2.49E-03 ± 1.19E-03 #	6.42E-03 ± 2.70E-03 * #	4.49E-04 ± 3.44E-05
Thermus	0.00E00 ± 0.00E00	0.00E00 ± 0.00E00	6.89E-05 ± 5.65E-05 #	1.49E-02 ± 1.49E-02 * #	0.00E00 ± 0.00E00	0.00E00 ± 0.00E00
Tuberibacillus	0.00E00 ± 0.00E00	0.00E00 ± 0.00E00	1.18E-02 ± 5.86E-03 * #	5.43E-05 ± 5.43E-05	1.40E-02 ± 1.22E-02 * #	2.84E-04 ± 2.84E-04

Supplementary Table S5 Relative abundance of differential abundant bacterial (16S rRNA V3-V4) genera in the subtypes of management residues (M1-M4) as compared to the two subtypes of peat-based substrates (P1 and P2) that represent at least 1% in one of the subtypes. Asterisks indicate a significant difference in the relative abundance as compared to subtype P1 of peat-based substrates. Hashtags indicate a significant difference in the relative abundance as compared to subtype P2 of peat-based substrates. P1 = pure peat-based substrates (n = 5); P2 = limed peat-based substrates (n = 5); M1 = grass clippings (n = 4); M2 = chopped heath (n = 4); M3 = forest sods (n = 2); M4 = woody fractions of composts (n = 2).

Genus	P1	P2	M1	M2	M3	M4
Achromobacter	9.11E-05 ± 6.67E-05	1.82E-05 ± 1.82E-05	1.49E-02 ± 9.00E-03 #	7.12E-05 ± 7.12E-05	0.00E00 ± 0.00E00	7.02E-03 ± 6.89E-03 #
Acinetobacter	0.00E00 ± 0.00E00	0.00E00 ± 0.00E00	4.63E-05 ± 3.00E-05	0.00E00 ± 0.00E00	0.00E00 ± 0.00E00	1.76E-02 ± 1.67E-02 * #
Actinomadura	5.31E-04 ± 3.74E-04	0.00E00 ± 0.00E00	4.74E-03 ± 3.44E-03 #	1.98E-03 ± 1.98E-03 #	0.00E00 ± 0.00E00	1.85E-02 ± 1.67E-02 #
Allorhizobium	1.72E-04 ± 1.43E-04	4.86E-05 ± 3.42E-05	1.26E-02 ± 5.15E-03 #	1.01E-02 ± 4.00E-03	0.00E00 ± 0.00E00	3.01E-02 ± 2.92E-02 #
Anaerolinea	5.88E-05 ± 5.88E-05	0.00E00 ± 0.00E00	0.00E00 ± 0.00E00	2.65E-03 ± 2.65E-03 #	0.00E00 ± 0.00E00	2.01E-05 ± 2.01E-05
Arachidicoccus	3.04E-04 ± 3.04E-04	1.76E-02 ± 1.76E-02	2.07E-03 ± 1.73E-03	0.00E00 ± 0.00E00 #	0.00E00 ± 0.00E00	5.11E-03 ± 5.11E-03
Bacillus	1.94E-04 ± 1.94E-04	2.52E-04 ± 2.37E-04	2.51E-03 ± 1.03E-03	4.47E-03 ± 3.93E-03	3.17E-04 ± 3.17E-04	1.14E-02 ± 7.57E-03 #
Bauldia	1.68E-03 ± 1.62E-03	1.80E-02 ± 7.74E-03	2.86E-04 ± 2.86E-04	2.89E-03 ± 1.61E-03	0.00E00 ± 0.00E00	0.00E00 ± 0.00E00 #
Brevibacterium	6.75E-05 ± 6.75E-05	0.00E00 ± 0.00E00	1.84E-03 ± 1.80E-03 #	1.14E-05 ± 1.14E-05	0.00E00 ± 0.00E00	6.90E-04 ± 2.82E-04 #
Cellvibrio	2.30E-04 ± 1.66E-04	0.00E00 ± 0.00E00	2.31E-03 ± 1.18E-03 #	2.32E-03 ± 2.22E-03 #	0.00E00 ± 0.00E00	7.19E-03 ± 6.85E-04 #
Chthoniobacter	1.01E-04 ± 1.01E-04	0.00E00 ± 0.00E00	1.57E-04 ± 1.09E-04	1.02E-02 ± 6.18E-03 #	4.48E-03 ± 1.76E-03	3.81E-05 ± 1.81E-05
Cytophaga	3.07E-04 ± 1.96E-04	0.00E00 ± 0.00E00	2.31E-03 ± 9.71E-04 #	1.68E-02 ± 1.12E-02 #	0.00E00 ± 0.00E00	5.37E-03 ± 5.37E-03 #
Dokdonella	1.61E-03 ± 1.61E-03	1.89E-02 ± 9.72E-03	1.33E-04 ± 1.33E-04	2.50E-03 ± 2.50E-03	0.00E00 ± 0.00E00	0.00E00 ± 0.00E00 #
Domibacillus	2.25E-04 ± 2.25E-04	0.00E00 ± 0.00E00	0.00E00 ± 0.00E00	0.00E00 ± 0.00E00	0.00E00 ± 0.00E00	1.07E-03 ± 3.57E-04 #
Enterobacter	0.00E00 ± 0.00E00	0.00E00 ± 0.00E00	2.08E-02 ± 1.13E-02 * #	0.00E00 ± 0.00E00	0.00E00 ± 0.00E00	1.02E-03 ± 7.62E-05 #
Filomicrobium	3.70E-04 ± 2.35E-04	0.00E00 ± 0.00E00	0.00E00 ± 0.00E00	0.00E00 ± 0.00E00	0.00E00 ± 0.00E00	3.32E-03 ± 3.15E-03 #
Flavobacterium	1.15E-03 ± 6.83E-04	0.00E00 ± 0.00E00	2.18E-02 ± 1.29E-02 #	2.15E-03 ± 1.64E-03 #	7.59E-05 ± 3.32E-05	1.91E-02 ± 1.52E-02 #
Geobacillus	1.53E-04 ± 1.53E-04	0.00E00 ± 0.00E00	0.00E00 ± 0.00E00	0.00E00 ± 0.00E00	0.00E00 ± 0.00E00	1.08E-02 ± 6.43E-03 #
Glutamicibacter	1.80E-04 ± 1.80E-04	0.00E00 ± 0.00E00	1.62E-04 ± 5.61E-05	0.00E00 ± 0.00E00	0.00E00 ± 0.00E00	1.28E-01 ± 1.27E-01 * #
Glycomyces	5.21E-04 ± 3.45E-04	0.00E00 ± 0.00E00	1.07E-04 ± 1.07E-04	0.00E00 ± 0.00E00	0.00E00 ± 0.00E00	1.60E-04 ± 1.60E-04 #
Granulicella	1.10E-01 ± 4.97E-02	4.07E-02 ± 3.40E-02	2.58E-02 ± 1.58E-02	1.73E-02 ± 8.36E-03	2.22E-02 ± 5.26E-03	0.00E00 ± 0.00E00 * #
Kribbella	8.62E-06 ± 8.62E-06	1.60E-02 ± 1.22E-02	0.00E00 ± 0.00E00 #	0.00E00 ± 0.00E00 #	0.00E00 ± 0.00E00	5.62E-05 ± 5.62E-05
Kurthia	0.00E00 ± 0.00E00	0.00E00 ± 0.00E00	0.00E00 ± 0.00E00	0.00E00 ± 0.00E00	0.00E00 ± 0.00E00	6.31E-02 ± 6.31E-02 * #
Lelliottia	0.00E00 ± 0.00E00	0.00E00 ± 0.00E00	1.04E-02 ± 6.38E-03 #	0.00E00 ± 0.00E00	0.00E00 ± 0.00E00	5.13E-03 ± 4.91E-03 #
Micropepsis	4.26E-03 ± 4.26E-03	4.62E-02 ± 2.31E-02	3.09E-04 ± 2.89E-04	1.25E-02 ± 1.15E-02	1.10E-03 ± 3.07E-04	0.00E00 ± 0.00E00 #
Nonomuraea	0.00E00 ± 0.00E00	0.00E00 ± 0.00E00	6.15E-02 ± 5.40E-02 * #	1.12E-05 ± 1.12E-05	0.00E00 ± 0.00E00	1.27E-02 ± 1.00E-02 * #
Occallatibacter	1.24E-01 ± 4.84E-02	7.72E-03 ± 4.35E-03	7.67E-04 ± 4.69E-04 *	3.01E-02 ± 2.15E-02	4.62E-02 ± 8.55E-03	9.35E-05 ± 9.35E-05
Ochrobactrum	0.00E00 ± 0.00E00	0.00E00 ± 0.00E00	1.24E-02 ± 9.57E-03 * #	1.87E-05 ± 1.87E-05	0.00E00 ± 0.00E00	1.99E-02 ± 1.38E-02 * #
Oerskovia	0.00E00 ± 0.00E00	0.00E00 ± 0.00E00	2.34E-02 ± 2.30E-02 * #	0.00E00 ± 0.00E00	0.00E00 ± 0.00E00	1.68E-03 ± 1.52E-03 #
Olivibacter	0.00E00 ± 0.00E00	0.00E00 ± 0.00E00	2.40E-04 ± 2.40E-04	0.00E00 ± 0.00E00	0.00E00 ± 0.00E00	1.41E-02 ± 3.74E-03 * #
Ornithinicoccus	0.00E00 ± 0.00E00	0.00E00 ± 0.00E00	0.00E00 ± 0.00E00	0.00E00 ± 0.00E00	0.00E00 ± 0.00E00	1.27E-04 ± 1.27E-04 #
Pedobacter	1.16E-03 ± 7.57E-04	8.15E-05 ± 8.15E-05	5.74E-03 ± 4.65E-03	0.00E00 ± 0.00E00	0.00E00 ± 0.00E00	1.53E-02 ± 1.52E-02 #
Planifilum	4.28E-05 ± 4.28E-05	0.00E00 ± 0.00E00	0.00E00 ± 0.00E00	0.00E00 ± 0.00E00	0.00E00 ± 0.00E00	5.49E-03 ± 5.14E-03 #
Promicromonospora	1.56E-04 ± 9.99E-05	2.51E-05 ± 2.51E-05	6.52E-03 ± 4.80E-03 #	0.00E00 ± 0.00E00	0.00E00 ± 0.00E00	1.78E-02 ± 8.69E-03 #
Pseudomonas	3.72E-03 ± 2.62E-03	2.61E-04 ± 1.69E-04	2.39E-02 ± 2.00E-02 #	1.61E-02 ± 7.39E-03	1.59E-04 ± 1.59E-04	4.56E-02 ± 2.82E-02 #
Pseudonocardia	6.98E-05 ± 6.98E-05	1.86E-05 ± 1.86E-05	1.14E-03 ± 6.64E-04	8.51E-05 ± 8.51E-05	6.58E-04 ± 2.74E-04	3.40E-03 ± 3.14E-03 #
Pusillimonas	0.00E00 ± 0.00E00	0.00E00 ± 0.00E00	1.70E-05 ± 1.70E-05	0.00E00 ± 0.00E00	0.00E00 ± 0.00E00	7.01E-05 ± 7.01E-05 #
Rahnella	0.00E00 ± 0.00E00	0.00E00 ± 0.00E00	1.24E-02 ± 7.29E-03 * #	6.06E-04 ± 5.40E-04	0.00E00 ± 0.00E00	5.91E-03 ± 5.91E-03 #
Sphaerobacter	2.41E-04 ± 2.41E-04	0.00E00 ± 0.00E00	0.00E00 ± 0.00E00	6.86E-04 ± 6.86E-04	0.00E00 ± 0.00E00	2.09E-03 ± 2.02E-03 #
Sphingobacterium	1.45E-04 ± 9.21E-05	0.00E00 ± 0.00E00	1.22E-02 ± 7.46E-03 #	4.12E-05 ± 4.12E-05	0.00E00 ± 0.00E00	1.62E-02 ± 1.63E-03 #
Staphylococcus	0.00E00 ± 0.00E00	0.00E00 ± 0.00E00	6.57E-03 ± 6.22E-03 * #	0.00E00 ± 0.00E00	0.00E00 ± 0.00E00	7.07E-03 ± 7.07E-03 * #
Stenotrophomonas	3.10E-05 ± 3.10E-05	0.00E00 ± 0.00E00	1.48E-02 ± 1.36E-02 #	0.00E00 ± 0.00E00	1.54E-04 ± 1.54E-04	1.93E-02 ± 1.81E-02 #
Streptacidiphilus	0.00E00 ± 0.00E00	7.06E-04 ± 4.33E-04	2.30E-02 ± 2.30E-02 *	1.40E-03 ± 1.40E-03	7.48E-03 ± 4.94E-03	0.00E00 ± 0.00E00
SWB02	4.62E-04 ± 4.62E-04	1.99E-02 ± 8.44E-03	0.00E00 ± 0.00E00 #	5.39E-04 ± 5.39E-04	0.00E00 ± 0.00E00	0.00E00 ± 0.00E00 #
Thermobispora	0.00E00 ± 0.00E00	0.00E00 ± 0.00E00	0.00E00 ± 0.00E00	0.00E00 ± 0.00E00	0.00E00 ± 0.00E00	5.15E-03 ± 4.47E-03 * #
Thermocrispum	4.05E-05 ± 4.05E-05	0.00E00 ± 0.00E00	4.24E-05 ± 4.24E-05	0.00E00 ± 0.00E00	0.00E00 ± 0.00E00	1.69E-03 ± 1.69E-03 #
Thermogemmatispora	0.00E00 ± 0.00E00	0.00E00 ± 0.00E00	0.00E00 ± 0.00E00	1.80E-02 ± 1.80E-02 #	0.00E00 ± 0.00E00	0.00E00 ± 0.00E00
Thermomonospora	1.80E-05 ± 1.80E-05	0.00E00 ± 0.00E00	5.73E-05 ± 5.73E-05	1.08E-04 ± 1.08E-04	0.00E00 ± 0.00E00	4.49E-03 ± 4.07E-03 #
Thermopolyspora	0.00E00 ± 0.00E00	0.00E00 ± 0.00E00	1.48E-05 ± 1.48E-05	0.00E00 ± 0.00E00	0.00E00 ± 0.00E00	8.41E-03 ± 8.04E-03 * #
Thermus	0.00E00 ± 0.00E00	0.00E00 ± 0.00E00	0.00E00 ± 0.00E00	0.00E00 ± 0.00E00	0.00E00 ± 0.00E00	1.24E-02 ± 1.24E-02 * #
Tuberibacillus	0.00E00 ± 0.00E00	0.00E00 ± 0.00E00	6.14E-04 ± 5.76E-04 #	1.67E-04 ± 9.91E-05	0.00E00 ± 0.00E00	1.36E-02 ± 3.26E-04 * #

Supplementary Table S6 Relative abundance of differential abundant fungal (ITS2) genera in the subtypes of composts (C1-C4) as compared to the two subtypes of peat-based substrates (P1 and P2). Asterisks indicate a significant difference in the relative abundance as compared to subtype P1 of peat-based substrates. Hashtags indicate a significant difference in the relative abundance as compared to subtype P2 of peat-based substrates. P1 = pure peat-based substrates (n = 5); P2 = limed peat-based substrates (n = 5); C1 = green composts (n = 7); C2 = VFG composts (n = 3); C3 = woody composts (n = 4); C4 = peat composts (n = 2); M1 = grass clippings (n = 4); M2 = chopped heath (n = 4); M3 = forest sods (n = 2); M4 = woody fractions of composts (n = 2).

Genus	P1	P2	C1	C2	C3	C4
Achroceratosphaeria	0.00E00 ± 0.00E00	0.00E00 ± 0.00E00	0.00E00 ± 0.00E00	7.87E-05 ± 7.87E-05	1.81E-02 ± 1.79E-02 * #	0.00E00 ± 0.00E00
Basidiodendron	4.34E-02 ± 3.94E-02	3.76E-04 ± 1.81E-04	0.00E00 ± 0.00E00 *	5.43E-06 ± 5.43E-06	0.00E00 ± 0.00E00 *	4.08E-05 ± 4.08E-05
Coprinus	0.00E00 ± 0.00E00	1.61E-04 ± 1.61E-04	6.03E-02 ± 3.81E-02 *	1.17E-04 ± 1.17E-04	1.89E-02 ± 1.67E-02 *	2.72E-05 ± 2.72E-05
Issatchenkia	0.00E00 ± 0.00E00	0.00E00 ± 0.00E00	1.25E-05 ± 1.25E-05	0.00E00 ± 0.00E00	4.43E-02 ± 4.43E-02 * #	0.00E00 ± 0.00E00
Kluyveromyces	0.00E00 ± 0.00E00	0.00E00 ± 0.00E00	6.83E-06 ± 6.83E-06	8.54E-05 ± 6.26E-05	4.05E-02 ± 4.04E-02 * #	0.00E00 ± 0.00E00
Leucocoprinus	1.75E-02 ± 1.75E-02	4.83E-04 ± 4.83E-04	0.00E00 ± 0.00E00 *	0.00E00 ± 0.00E00	0.00E00 ± 0.00E00	0.00E00 ± 0.00E00
Mucor	1.39E-04 ± 3.94E-05	0.00E00 ± 0.00E00	9.58E-03 ± 7.77E-03	2.11E-03 ± 1.81E-03	4.28E-03 ± 3.85E-03 #	9.95E-05 ± 9.31E-06
Pichia	0.00E00 ± 0.00E00	0.00E00 ± 0.00E00	2.62E-04 ± 1.84E-04	0.00E00 ± 0.00E00	2.21E-02 ± 2.21E-02 * #	0.00E00 ± 0.00E00
Plectosphaerella	0.00E00 ± 0.00E00	2.11E-04 ± 1.50E-04	0.00E00 ± 0.00E00	6.52E-05 ± 6.52E-05	6.82E-02 ± 6.80E-02 *	0.00E00 ± 0.00E00
Thermothielavioides	1.94E-04 ± 1.86E-04	0.00E00 ± 0.00E00	1.69E-05 ± 1.69E-05	2.19E-05 ± 2.19E-05	4.12E-03 ± 3.86E-03 #	4.08E-04 ± 4.08E-04

Supplementary Table S7 Relative abundance of differential abundant fungal (ITS2) genera in the subtypes of management residues (M1-M4) as compared to the two subtypes of peat-based substrates (P1 and P2). Asterisks indicate a significant difference in the relative abundance as compared to subtype P1 of peat-based substrates. Hashtags indicate a significant difference in the relative abundance as compared to subtype P2 of peat-based substrates. P1 = pure peat-based substrates (n = 5); P2 = limed peat-based substrates (n = 5); M1 = grass clippings (n = 4); M2 = chopped heath (n = 4); M3 = forest sods (n = 2); M4 = woody fractions of composts (n = 2).

Genus	P1	P2	M1	M2	M3	M4
Achroceratosphaeria	0.00E00 ± 0.00E00	0.00E00 ± 0.00E00	1.43E-04 ± 1.43E-04	5.51E-03 ± 5.23E-03 #	0.00E00 ± 0.00E00	5.16E-02 ± 5.16E-02 * #
Acidomelania	9.14E-06 ± 9.14E-06	0.00E00 ± 0.00E00	2.14E-04 ± 2.04E-04	1.06E-02 ± 5.76E-03 #	4.06E-03 ± 3.83E-03	0.00E00 ± 0.00E00
Basidiodendron	4.34E-02 ± 3.94E-02	3.76E-04 ± 1.81E-04	0.00E00 ± 0.00E00 *	2.71E-03 ± 2.38E-03	0.00E00 ± 0.00E00	0.00E00 ± 0.00E00
Ceratobasidium	0.00E00 ± 0.00E00	0.00E00 ± 0.00E00	3.77E-04 ± 3.65E-04	1.92E-02 ± 1.21E-02 #	5.56E-04 ± 1.63E-04	0.00E00 ± 0.00E00
Clitopilus	6.96E-05 ± 5.67E-05	5.58E-02 ± 5.53E-02	2.84E-06 ± 2.84E-06	0.00E00 ± 0.00E00 #	9.43E-05 ± 9.43E-05	0.00E00 ± 0.00E00
Constantinomyces	2.01E-05 ± 2.01E-05	0.00E00 ± 0.00E00	1.63E-05 ± 9.39E-06	1.56E-02 ± 1.06E-02 #	1.85E-03 ± 1.85E-03	0.00E00 ± 0.00E00
Coprinus	0.00E00 ± 0.00E00	1.61E-04 ± 1.61E-04	7.73E-05 ± 4.15E-05	1.74E-05 ± 1.74E-05	1.96E-05 ± 1.96E-05	4.00E-02 ± 2.04E-02 *
Cystodendron	0.00E00 ± 0.00E00	0.00E00 ± 0.00E00	2.96E-05 ± 1.72E-05	9.37E-03 ± 5.49E-03 #	1.06E-02 ± 1.06E-02	0.00E00 ± 0.00E00
Exophiala	0.00E00 ± 0.00E00	0.00E00 ± 0.00E00	0.00E00 ± 0.00E00	7.91E-03 ± 6.81E-03 #	2.11E-03 ± 2.11E-03	0.00E00 ± 0.00E00
Fimetariella	5.07E-02 ± 4.56E-02	3.95E-04 ± 2.54E-04	0.00E00 ± 0.00E00 *	0.00E00 ± 0.00E00	2.24E-04 ± 2.24E-04	0.00E00 ± 0.00E00
Meliniomyces	4.66E-01 ± 1.57E-01	7.49E-02 ± 2.97E-02	0.00E00 ± 0.00E00 * #	1.43E-02 ± 7.96E-03	2.74E-02 ± 1.78E-02	0.00E00 ± 0.00E00
Mucor	1.39E-04 ± 3.94E-05	0.00E00 ± 0.00E00	4.39E-03 ± 3.55E-03	9.52E-03 ± 7.98E-03 #	1.91E-02 ± 1.90E-02	5.17E-03 ± 2.88E-03
Myxocephala	0.00E00 ± 0.00E00	0.00E00 ± 0.00E00	3.83E-05 ± 3.83E-05	1.40E-02 ± 1.17E-02 #	9.43E-05 ± 9.43E-05	0.00E00 ± 0.00E00
Rasamsonia	5.11E-05 ± 3.58E-05	0.00E00 ± 0.00E00	3.30E-04 ± 3.11E-04	5.43E-03 ± 4.63E-03 #	2.36E-05 ± 2.36E-05	0.00E00 ± 0.00E00
Schwanniomyces	0.00E00 ± 0.00E00	0.00E00 ± 0.00E00	1.01E-02 ± 1.01E-02 *	2.19E-04 ± 2.15E-04	1.16E-04 ± 1.00E-04	0.00E00 ± 0.00E00
Sistotrema	0.00E00 ± 0.00E00	0.00E00 ± 0.00E00	3.74E-04 ± 3.67E-04	7.94E-03 ± 6.70E-03 #	5.66E-04 ± 2.04E-04	0.00E00 ± 0.00E00
Sugiyamaella	1.46E-02 ± 8.65E-03	4.41E-04 ± 2.77E-04	0.00E00 ± 0.00E00 *	2.76E-03 ± 2.76E-03	1.89E-03 ± 9.93E-04	0.00E00 ± 0.00E00
Sympodiella	3.75E-04 ± 1.95E-04	0.00E00 ± 0.00E00	3.85E-05 ± 3.85E-05	8.08E-03 ± 3.84E-03 #	3.55E-02 ± 2.16E-02	0.00E00 ± 0.00E00
Sympoventuria	1.64E-05 ± 1.64E-05	0.00E00 ± 0.00E00	1.38E-04 ± 1.38E-04	7.84E-03 ± 4.36E-03 #	5.73E-03 ± 5.73E-03	0.00E00 ± 0.00E00
Talaromyces	3.27E-06 ± 3.27E-06	1.72E-03 ± 1.72E-03	8.11E-02 ± 7.38E-02 *	2.50E-03 ± 2.19E-03	2.50E-03 ± 2.09E-03	6.97E-05 ± 6.97E-05
Thermothielavioides	1.94E-04 ± 1.86E-04	0.00E00 ± 0.00E00	8.55E-04 ± 7.51E-04	2.75E-02 ± 1.91E-02 #	1.14E-04 ± 1.14E-04	2.50E-04 ± 2.50E-04
Westerdykella	0.00E00 ± 0.00E00	4.17E-04 ± 4.17E-04	7.70E-03 ± 7.70E-03 *	6.35E-04 ± 6.06E-04	3.42E-04 ± 3.42E-04	0.00E00 ± 0.00E00
Wickerhamomyces	0.00E00 ± 0.00E00	0.00E00 ± 0.00E00	9.27E-02 ± 9.27E-02 * #	5.04E-04 ± 4.51E-04	2.28E-04 ± 2.28E-04	0.00E00 ± 0.00E00

Supplementary Table S8 Mean relative abundances ± se of known antagonistic genera (*Bacillus*, *Burkholderia*, *Paenibacillus*, *Pseudomonas*, *Serratia*, *Streptomyces*, *Penicillium* and *Trichoderma*) in the subtypes of the three types of biomass. Asterisks indicate a significant difference in the relative abundance as compared to pure peat-based substrates (P1). Hashtag indicates a significant difference in the relative abundance as compared to limed peat-based substrates (P2). P1 = pure peat-based substrates (n = 5); P2 = limed peat-based substrates (n = 5); C1 = green composts (n = 7); C2 = VFG composts (n = 3); C3 = woody composts (n = 4); C4 = peat composts (n = 2); M1 = grass clippings (n = 4); M2 = chopped heath (n = 4); M3 = forest sods (n = 2); M4 = woody fractions of composts (n = 2).

Genus	P1	P2	C1	C2	C3	C4	M1	M2	M3	M4
Bacillus	1.94E-04 ± 1.94E-04	2.52E-04 ± 2.37E-04	2.30E-02 ± 5.13E-03 #	1.76E-02 ± 5.22E-03 #	5.36E-02 ± 3.22E-02 * #	1.66E-02 ± 1.45E-02	2.51E-03 ± 1.03E-03	4.47E-03 ± 3.93E-03	3.17E-04 ± 3.17E-04	1.14E-02 ± 7.57E-03 #
Burkholderia	4.95E-03 ± 2.64E-03	3.96E-03 ± 2.98E-03	0.00E00 ± 0.00E00 * #	0.00E00 ± 0.00E00 *,#	5.14E-04 ± 3.25E-04	0.00E00 ± 0.00E00	3.14E-02 ± 2.89E-02	1.38E-02 ± 5.63E-03	5.99E-02 ± 4.31E-02	1.58E-03 ± 1.58E-03
Paenibacillus	0.00E00 ± 0.00E00	0.00E00 ± 0.00E00	4.63E-03 ± 1.32E-03 * #	2.76E-03 ± 1.41E-03 #	5.01E-03 ± 1.48E-03 * #	3.69E-03 ± 3.69E-03	1.51E-03 ± 5.67E-04 #	0.00E00 ± 0.00E00	0.00E00 ± 0.00E00	3.52E-03 ± 2.41E-03 * #
Pseudomonas	3.72E-03 ± 2.62E-03	2.61E-04 ± 1.69E-04	1.58E-02 ± 2.87E-03 #	4.53E-02 ± 2.99E-02 #	4.92E-03 ± 2.52E-03 #	8.13E-03 ± 7.00E-03	2.39E-02 ± 2.00E-02 #	1.61E-02 ± 7.39E-03	1.59E-04 ± 1.59E-04	4.56E-02 ± 2.82E-02 #
Serratia	5.47E-04 ± 4.32E-04	0.00E00 ± 0.00E00	2.41E-05 ± 2.41E-05	2.71E-05 ± 2.71E-05	0.00E00 ± 0.00E00 *	0.00E00 ± 0.00E00	9.39E-03 ± 5.34E-03 #	5.53E-05 ± 5.53E-05	1.21E-04 ± 1.21E-04	2.19E-03 ± 9.97E-04 #
Streptomyces	6.04E-04 ± 3.42E-04	2.70E-02 ± 1.56E-02	3.25E-02 ± 1.05E-02	3.44E-02 ± 1.94E-02	2.35E-02 ± 1.21E-02	2.01E-02 ± 1.44E-04	2.22E-01 ± 1.24E-01	7.12E-04 ± 2.11E-04	1.80E-03 ± 1.80E-03	6.55E-02 ± 3.09E-02
Penicillium	1.58E-03 ± 7.07E-04	1.73E-03 ± 9.82E-04	7.01E-03 ± 5.34E-03	1.49E-03 ± 1.33E-03	2.23E-03 ± 1.60E-03	3.04E-03 ± 5.19E-04	7.76E-03 ± 3.13E-03	7.34E-04 ± 4.14E-04	1.48E-02 ± 1.36E-02	0.00E00 ± 0.00E00
Trichoderma	5.27E-03 ± 3.37E-03	5.97E-02 ± 5.42E-02	1.64E-02 ± 9.84E-03	2.02E-02 ± 1.13E-02	1.23E-02 ± 7.24E-03	2.76E-03 ± 2.33E-04	6.45E-03 ± 5.68E-03	9.82E-02 ± 8.15E-02	5.20E-02 ± 3.35E-02	1.87E-04 ± 1.39E-04

Supplementary Table S9 Genera that were significantly more abundant in at least on of the subtypes of composts and management residues compared to peat-based substrates and that have been found in literature to include (A) biocontrol agents and/or (B) plant-growth promoting species.

A.

Genus	Species/strain	Pathogen	Plant	References
Achromobacter	*Achromobacter insolitus*	*Pythium aphanidermatum*	Cucumber	Yuliar et al. (2021)
	Achromobacter xylosoxidans	*Fusarium solani*	Common bean	Mohamadpoor et al. (2022)
		Fusarium oxysporum	Tomato	Moretti et al. (2008)
		Fusarium oxysporum, Fusarium solani	Melon	Dhaouadi et al. (2019)
Acinetobacter	*Acinetobacter lwoffii*	*Botrytis cinerea*	Grapevine	Trotel-Aziz et al. (2008)
	Acinetobacter sp.	*Ralstonia solanacearum*	Tomato	Xue et al. (2009)
		Fusarium oxysporum	Cucumber	Du et al. (2017)
Actinomadura	*Actinomadura sp.*	*Phytophthora cinnamomi*	Avocado	You et al. (1996)
Allorhizobium	*Allorhizobium vitis*	*Allorhizobium vitis (Ti)*	Grapevine	Kawaguchi & Noutoshi (2022)
		Allorhizobium vitis (Ti)	Grapevine	Xi et al. (2022)
Bacillus	*Bacillus amyloliquefaciens*	*Pythium schmitthenneri*	Olive trees	Legrifi et al. (2022)
		Xanthomonas axonopodiss	Soybean	Preecha et al. (2010)
		Xanthomonas arboricola, Pseudomonas syringae	Walnut	Dimkić et al. (2013, 2017)
		Fusarium oxysporum	Cucumber	Du et al. (2017)
		Ralstonia solanacearum	Pepper	Eljounaidi et al. (2016)
	Bacillus circulans	*Fusarium oxysporum*	Tomato	Hsieh et al. (2008)
	Bacillus licheniformis	*Fusarium oxysporum*	Cucumber	Du et al. (2017)
	Bacillus pumilus	*Rhizoctonia solani, Pythium aphanidermatum, Sclerotium rolfsii*	Soybean, peanut, tomato, watermelon, cowpea	de Melo et al. (2009)
	Bacillus subtilis	*Pythium ultimum*	Bean	Ongena et al. (2005)
		Colletotrichum gloeosporioides	Orchid	Thasana et al. (2010)
		Fusarium oxysporum	Cucumber	Cao et al. (2012); Falardeau et al. (2013)
		Fusarium oxysporum, Rosellinia necatrix	Tomato, avocado	Cazorla et al. (2007)
		Rhizoctonia solani, Phomopsis sp.	Cucumber, tomato	Kita et al. (2005)
		Xanthomonas campestris, Pectobacterium carotovorum	Melon	Zeriouh et al. (2011)
		Verticillium dahliae, Fusarium oxysporum	Tomato	Tsolakidou et al. (2019)
		Rhizosctonia solani	Lettuce, carrot, cucumber, tomato	Grosch et al. (2004)

		Verticillium dahliae	Eggplant	Eljounaidi et al. (2016)
		Fusarium, Rhizoctonia, Alternaria, Aspergillus	Beans, peas, soybeans	Fravel (2005)
		Fusarium sp., Rhizoctonia sp.	Shade and forest trees, ornamentals, shrubs	Fravel (2005)
	Bacillus tequilensis	*Ganoderma boninense*	Oil palm	Chin et al. (2017)
	Bacillus thuringiensis	*Colletotrichum gloeosporioides*	Fruits	Kim et al. (2004)
	Bacillus velezensis	*Phytophthora capsici, P. citricola, P. palmivora, P. cinnamomi*	Lettuce	Syed-Ab-Rahman et al. (2018)
		Botrytis cinerea	Tomato	Li et al. (2022)
	Bacillus sp.	*Fusarium graminearum, Rhizoctonia solani, Fusarium oxysporum, Pythium irregulare, Botrytis cinerea*	Watermelon	Zhao et al. (2014)
Brevibacterium	*Brevibacterium iodinum*	*Stemphylium lycopersici*	Pepper	Son et al. (2014)
Flavobacterium	*Flavobacterium johnsoniae*	*Phytophthora capsici*	Pepper	Sang & Kim (2012)
Geobacillus	*Geobacillus thermodenitrificans*	*Phytophthora capsici*	Pepper	Sánchez San Fulgencio et al. (2018)
Mucor	*Mucor moelleri*	*Athelia rolfsii, Colletotrichum gloeosporiodes*	Tomato	Nartey et al. (2022)
Ochrobactrum	*Ochrobactrum anthropi*	*Phellinus noxius*	Tea plants	Chakraborty et al. (2009)
	Ochrobactrum ciceri	*Macrophomina phaseolina*	Mungbean	Shoaib et al. (2020)
		Botrytis cinerea	Lilium	Priyanka & Nakkeeran (2019)
	Ochrobactrum sp.	*Verticillium dahliae, Fusarium oxysporum*	Tomato	Tsolakidou et al. (2019)
Paenibacillus	*Paenibacillus sp.*	*Verticillium dahliae*	Eggplant	Eljounaidi et al. (2016)
Pichia	*Pichia guillermondii*	*Ralstonia solanacearum*	Tomato	Nguyen et al. (2011)
Pseudomonas	*Pseudomonas aeruginosa*	*Xanthomonas campestris, Ralstonia solanacearum*	Tomato	Hariprasad et al. (2014)
	Pseudomonas cepacia	*Rhizoctonia solani*	Lettuce	Grosch et al. (2004)
	Pseudomonas chlororaphis	*Sclerotinia sclerotiorum*	Lettuce, carrots	Nerek et al. (2022)
		Phytium, sp., Rhizoctonia solani, Fusarium oxysporum	Vegetables, ornamentals	Fravel (2005)
		Coletotrichum lagenarium, Pythium ultimum, Sclerotinia sclerotiorum, Fusarium oxysporum, Carposina sasakii, Rhizoctonia solani	Green pepper	Sandhya et al. (2010)
	Pseudomonas fluorescens	*Pythium ultimum, Gaeumannomyces graminis, Fusarium oxysporum , Phomopsis sclerotioides, Thielaviopsis basicola and Rhizoctonia solani*	Cucumber	Maurhofer et al. (1994)
		Ralstonia solanacearum	Eggplant	Eljounaidi et al. (2016)

		Verticillium dahlia	Olive	Eljounaidi et al. (2016)
	Pseudomonas sp.	*Fusarium oxysporum*	Carnation	Van Peer & Schippers (1992)
		Phytium sp.	Cucumber	Chen et al. (2012)
Rahnella	*Rahnella aquatilis*	*Colletotrichum gloeosporioides*	Liriodendron chinense × tulipifera	Kong et al. (2020)
		Xanthomonas campestris	Tomato	El-Hendawy et al. (2005)
		Xanthomonas axonopodis	Common bean	Sallam (2011)
		Erwinia amylovora	Apple	Abo-Elyousr et al. (2010)
		Xanthomonas campestris	Cihinese cabbage	Fei et al. (2019)
Serratia	*Serratia marcescens*	*Fusarium oxysporum*	Banana	Eljounaidi et al. (2016)
Talaromyces	*Talaromyces apiculatus*	*Ganoderma boninense*	Oil palm	Goh et al. (2020)
	Talaromyces flavus	*Sclerotium rolfsii, Verticillium dahliae*	Common bean	Madi et al. (1997)
		Sclerotinia sclerotiorum	Sunflower	McLaren et al. (1994)
		Verticillium albo-atrum	Tomato	Naraghi et al. (2010)
		Sclerotium rolfsii	Mungbean	Chakraborty et al. (2012); Dethoup et al. (2007); Sunar & Dey (2012)
	Talaromyces pinophilus	*Pythium aphanidermatum, Rhizoctonia solani*	Cucumber	Kazerooni et al. (2019)
	Talaromyces purpurogenus	*Fusarium oxysporum*	Bitter gourd	Tian et al. (2022)
	Talaromyces variabilis	*Pythium aphanidermatum*	Cucumber, tomato	Halo et al. (2019)
	Talaromyces sp.	*Fusarium oxysporum*	Bitter gourd	Tian et al. (2021)

B.

Genus	Species/strain	Plant	Reference
Acinetobacter	*Acinetobacter calcoaceticus*	Cucumber	Kang et al. (2009, 2012)
Bacillus	*Bacillus amyloliquefaciens*	Tomato	Gül et al. (2008)
	Bacillus aryabhattai	Soybean	Miljaković et al. (2020)
	Bacillus megaterium	Common bean, eggplant, pepper, tomato	Miljaković et al. (2020); Ortíz-Castro et al. (2008)
	Bacillus methylotrophicus	Lettuce, muskmelon, soybean, vegetable mustard	Miljaković et al. (2020)
	Bacillus simplex	Eggplant, pepper, and tomato	Miljaković et al. (2020)
	Bacillus subtilis	Tomato, spinach, eggplant, pepper, cucumber, lettuce	Adesemoye et al. (2008); Miljaković et al. (2020); Yao et al. (2006)
Exophiala	*Exophiala sp.*	Cucumber	Khan et al. (2011)
Geobacillus	*Geobacillus thermodenitrificans*	Pepper	Sánchez San Fulgencio et al. (2018)

Glutamicibacter	*Glutamicibacter halophytocola*	Limonium sinense, tomato	Qin et al. (2018); Xiong et al. (2019)
Mucor	*Mucor moelleri*	Tomato	Nartey et al. (2022)
Ochrobactrum	*Ochrobactrum anthropi*	Tea plants	Chakraborty et al. (2009)
	Ochrobactrum sp.	Soybean	Yu et al. (2017)
Pichia	*Pichia sp.*	Lettuce	Nakayan et al. (2009)
Promicromonospora	*Promicromonospora sp.*	Tomato, cucumber	Kang et al. (2012, 2014)
Pseudomonas	*Pseudomonas aeruginosa*	Tomato, okra, spinach	Adesemoye et al. (2008)
	Pseudomonas aeruginosa	Tomato	Hariprasad et al. (2014)
	Pseudomonas fluorescens	Tomato	Choi et al. (2008)
	Pseudomonas putida	Soybean	Costa-Gutierrez et al. (2020)
	Pseudomonas putida	Canola, lettuce, tomato	Hall et al. (1996)
**Rahnella**	Rahnella sp.	Pea	Vyas et al., (2010)
**Staphylococcus**	Staphylococcus sp.	Strawberry	Ipek et al. (2014)
**Talaromyces**	Talaromyces apiculatus	Oil palm	Goh et al. (2020)
	Talaromyces flavus	Cucumber, tomato	Naraghi et al. (2012)
	Talaromyces flavus	Mungbean	Chakraborty et al. (2012)
	Talaromyces omanensis	Tomato	Halo et al. (2020)
	Talaromyces purpurogenus	Bitter gourd	Tian et al. (2022)

Supplementary Table S10 Mean relative abundances ± se of genera known to include human and/or plant pathogens present in the samples (*Escherichia*/*Shigella*, *Klebsiella* and *Enterobacter*) for the subtypes of the three types of biomass. Asterisks indicate a significant difference in the relative abundance as compared to pure peat-based substrates (P1). Hashtag indicates a significant difference in the relative abundance as compared to limed peat-based substrates (P2). P1 = pure peat-based substrates (n = 5); P2 = limed peat-based substrates (n = 5); C1 = green composts (n = 7); C2 = VFG composts (n = 3); C3 = woody composts (n = 4); C4 = peat composts (n = 2); M1 = grass clippings (n = 4); M2 = chopped heath (n = 4); M3 = forest sods (n = 2); M4 = woody fractions of composts (n = 2).

Genus	P1	P2	C1	C2	C3	C4	M1	M2	M3	M4
Enterobacter	0.00E00 ± 0.00E00	0.00E00 ± 0.00E00	1.12E-04 ± 1.12E-04 #	0.00E00 ± 0.00E00	0.00E00 ± 0.00E00	0.00E00 ± 0.00E00	2.08E-02 ± 1.13E-02 *#	0.00E00 ± 0.00E00	0.00E00 ± 0.00E00	1.02E-03 ± 7.62E-05 #
Escherichia/Shigella	2.55E-04 ± 2.55E-04	0.00E00 ± 0.00E00	3.62E-06 ± 3.62E-06	0.00E00 ± 0.00E00	0.00E00 ± 0.00E00	0.00E00 ± 0.00E00	6.70E-03 ± 4.46E-03 #	0.00E00 ± 0.00E00	0.00E00 ± 0.00E00	2.07E-04 ± 2.07E-04 #
Klebsiella	0.00E00 ± 0.00E00	0.00E00 ± 0.00E00	2.20E-05 ± 2.20E-05	0.00E00 ± 0.00E00	0.00E00 ± 0.00E00	0.00E00 ± 0.00E00	2.44E-03 ± 1.63E-03 #	0.00E00 ± 0.00E00	0.00E00 ± 0.00E00	1.28E-03 ± 9.02E-04 #

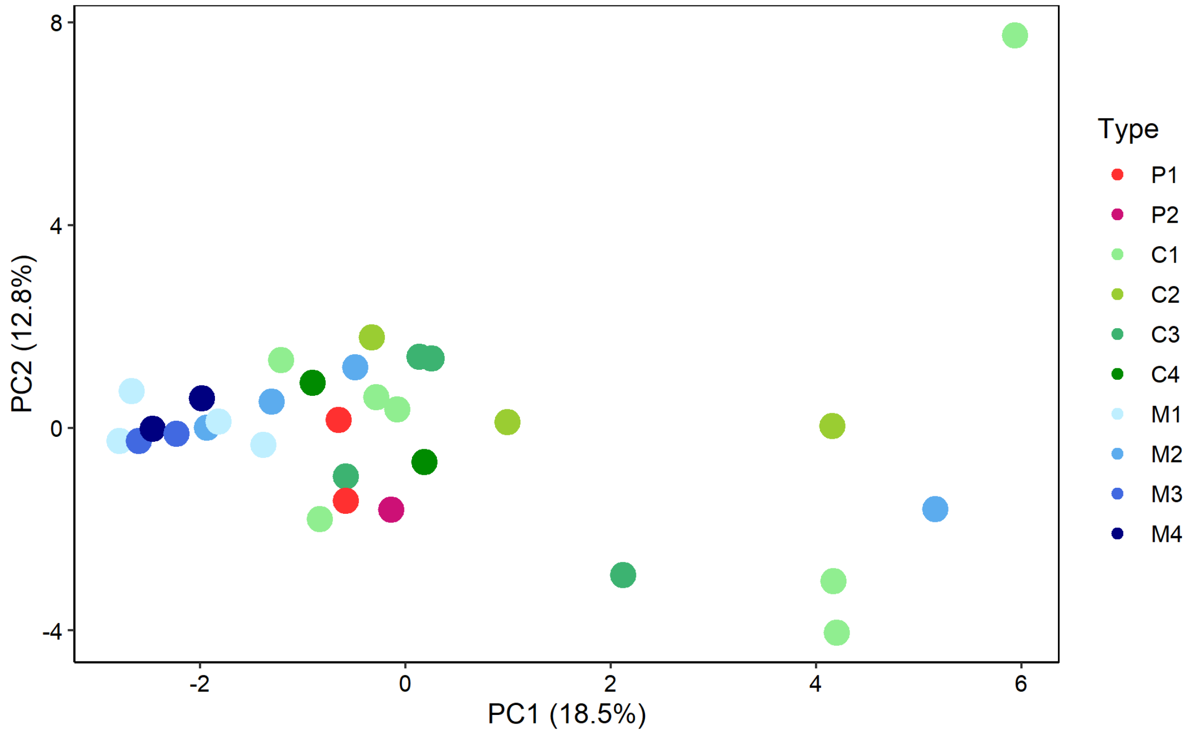

Supplementary Figure S5. Principal component analysis (PCA) on carbon source metabolization determined with Biolog Ecoplates with subtypes of peat-based substrates, composts and management residues. Colors indicate different subtypes of peat-based substrates (P1-P2), composts (C1-C4) and management residues (M1-M4). The first two principal components (PC) presented 18.5% and 12.8%, respectively, of the total variability in the dataset. P1 = pure peat-based substrates (n = 2); P2 = limed peat-based substrates (n = 1); C1 = green composts (n = 7); C2 = VFG composts (n = 3); C3 = woody composts (n = 4); C4 = peat composts (n = 2); M1 = grass clippings (n = 4); M2 = chopped heath (n = 4); M3 = forest sods (n = 2); M4 = woody fractions of composts (n = 2).

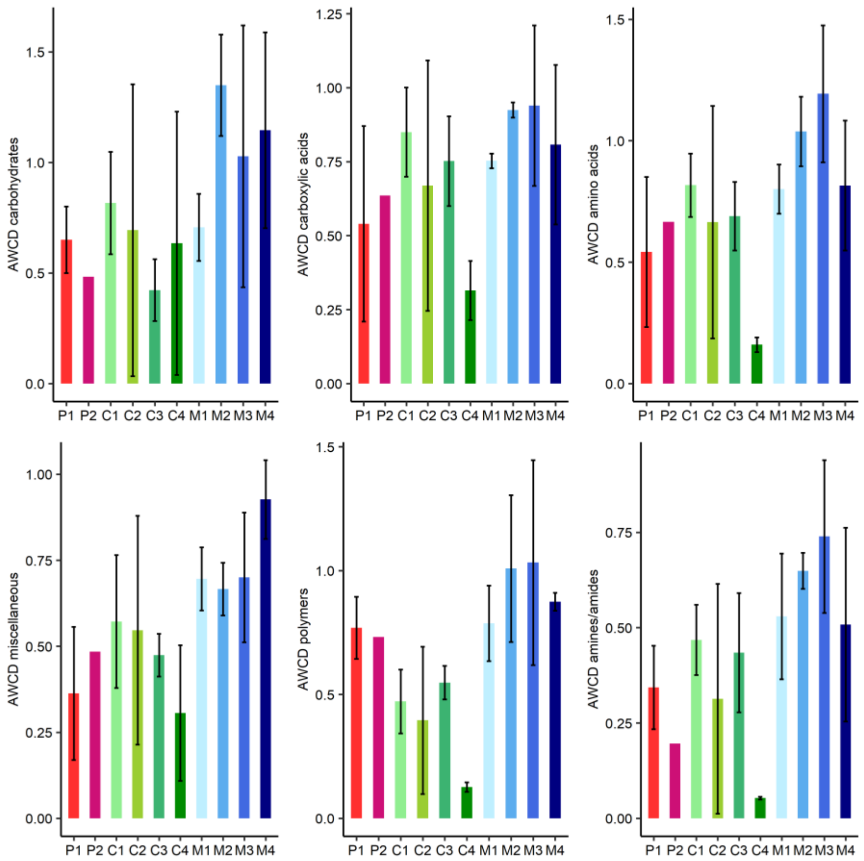

Supplementary Figure S6. Metabolic characteristics in subtypes of peat-based substrates (P1-P2), composts (C1-C4) and management residues (M1-M4). AWCD (average well color development) of carbohydrates, carboxylic acids, amino acids, miscellaneous C-sources, polymers and amines/amides. P1 = pure peat-based substrates (n = 2); P2 = limed peat-based substrates (n = 1); C1 = green composts (n = 7); C2 = VFG composts (n = 3); C3 = woody composts (n = 4); C4 = peat composts (n = 2); M1 = grass clippings (n = 4); M2 = chopped heath (n = 4); M3 = forest sods (n = 2); M4 = woody fractions of composts (n = 2).

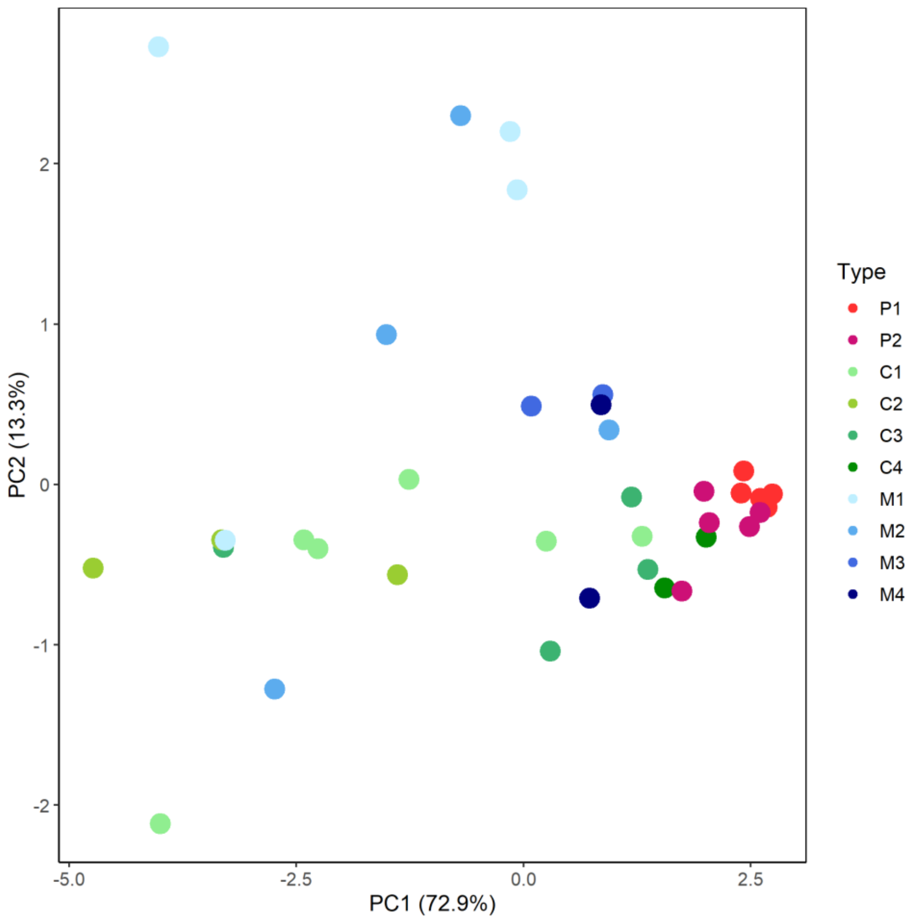

Supplementary Figure S7. Principal component analysis (PCA) on microbial biomass determined with PLFA analysis with subtypes of peat-based substrates, composts and management residues. Colors indicate different subtypes of peat-based substrates (P1-P2), composts (C1-C4) and management residues (M1-M4). The first two principal components (PC) presented 72.9% and 13.3%, respectively, of the total variability in the dataset. P1 = pure peat-based substrates (n = 5); P2 = limed peat-based substrates (n = 5); C1 = green composts (n = 7); C2 = VFG composts (n = 3); C3 = woody composts (n = 4); C4 = peat composts (n = 2); M1 = grass clippings (n = 4); M2 = chopped heath (n = 4); M3 = forest sods (n = 2); M4 = woody fractions of composts (n = 2).

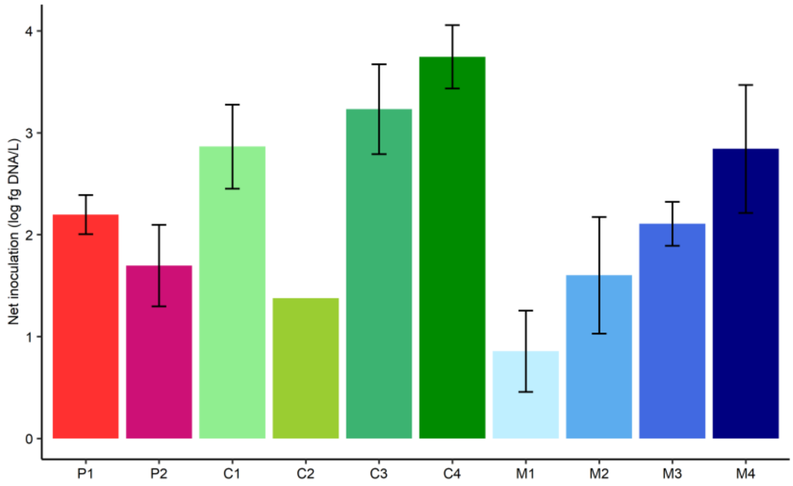

Supplementary Figure S8. Mean net inoculation ± se (log fg DNA/L) in the different subtypes of peat-based substrates, composts, and management residues. P1=pure peat-based substrates (n=5); P2=limed peat-based substrates (n=5); C1=green composts (n=7); C2=VFG composts (n=3); C3=woody composts (n=4); C4=peat composts (n=2); M1=grass clippings (n=4); M2=chopped heath (n=4); M3=forest sods (n=2); M4=woody fractions of composts (n=2).
